# Long Interleukin-22 Binding Protein Isoform-1 Is an Intracellular Activator of the Unfolded Protein Response

**DOI:** 10.3389/fimmu.2018.02934

**Published:** 2018-12-14

**Authors:** Paloma Gómez-Fernández, Andoni Urtasun, Adrienne W. Paton, James C. Paton, Francisco Borrego, Devin Dersh, Yair Argon, Iraide Alloza, Koen Vandenbroeck

**Affiliations:** ^1^Neurogenomiks Group, Department of Neuroscience, University of the Basque Country (UPV/EHU), Leioa, Spain; ^2^Achucarro Basque Center for Neuroscience, Leioa, Spain; ^3^Research for Infectious Diseases, Department of Molecular and Biomedical Science, University of Adelaide, Adelaide, SA, Australia; ^4^Biocruces Bizkaia Health Research Institute, Barakaldo, Spain; ^5^Basque Center for Transfusion and Human Tissues, Galdakao, Spain; ^6^IKERBASQUE, Basque Foundation for Science, Bilbao, Spain; ^7^Division of Cell Pathology, Children's Hospital of Philadelphia and Perelman School of Medicine, University of Pennsylvania, Philadelphia, PA, United States

**Keywords:** IL-22BP, IL-22, GRP78, GRP94, UPR, isoform, dendritic cells, exonization

## Abstract

The human *IL22RA2* gene co-produces three protein isoforms in dendritic cells [IL-22 binding protein isoform-1 (IL-22BPi1), IL-22BPi2, and IL-22BPi3]. Two of these, IL-22BPi2 and IL-22BPi3, are capable of neutralizing the biological activity of IL-22. The function of IL-22BPi1, which differs from IL-22BPi2 through an in-frame 32-amino acid insertion provided by an alternatively spliced exon, remains unknown. Using transfected human cell lines, we demonstrate that IL-22BPi1 is secreted detectably, but at much lower levels than IL-22BPi2, and unlike IL-22BPi2 and IL-22BPi3, is largely retained in the endoplasmic reticulum (ER). As opposed to IL-22BPi2 and IL-22BPi3, IL-22BPi1 is incapable of neutralizing or binding to IL-22 measured in bioassay or assembly-induced IL-22 co-folding assay. We performed interactome analysis to disclose the mechanism underlying the poor secretion of IL-22BPi1 and identified GRP78, GRP94, GRP170, and calnexin as main interactors. Structure-function analysis revealed that, like IL-22BPi2, IL-22BPi1 binds to the substrate-binding domain of GRP78 as well as to the middle domain of GRP94. Ectopic expression of wild-type GRP78 enhanced, and ATPase-defective GRP94 mutant decreased, secretion of both IL-22BPi1 and IL-22BPi2, while neither of both affected IL-22BPi3 secretion. Thus, IL-22BPi1 and IL-22BPi2 are *bona fide* clients of the ER chaperones GRP78 and GRP94. However, only IL-22BPi1 activates an unfolded protein response (UPR) resulting in increased protein levels of GRP78 and GRP94. Cloning of the *IL22RA2* alternatively spliced exon into an unrelated cytokine, IL-2, bestowed similar characteristics on the resulting protein. We also found that CD14^++^/CD16^+^ intermediate monocytes produced a higher level of *IL22RA2* mRNA than classical and non-classical monocytes, but this difference disappeared in immature dendritic cells (moDC) derived thereof. Upon silencing of *IL22RA2* expression in moDC, GRP78 levels were significantly reduced, suggesting that native *IL22RA2* expression naturally contributes to upregulating GRP78 levels in these cells. The *IL22RA2* alternatively spliced exon was reported to be recruited through a single mutation in the proto-splice site of a Long Terminal Repeat retrotransposon sequence in the ape lineage. Our work suggests that positive selection of IL-22BPi1 was not driven by IL-22 antagonism as in the case of IL-22BPi2 and IL-22BPi3, but by capacity for induction of an UPR response.

## Introduction

Interleukin-22 (IL-22) is an IL-10-type cytokine produced by a wide variety of immune cells including innate lymphoid cells, CD4^+^ Th17 and Th22 cells (1, 2) and neutrophils (3). It is crucially involved in diverse pathologic processes related to autoimmunity, epithelial cell characteristics and transformation, as well as to damage conferred by tumors. It is specifically involved in regulating the immunity of barrier surfaces (4). IL-22, which signals through a membrane receptor composed by the heterodimer IL-22R1/IL-10R2, has the unique feature among the IL-10 family cytokines of being recognized by a secreted receptor called IL-22 binding protein (IL-22BP), which is coded for by the *IL22RA2* gene. *IL22RA2* is expressed in different cells from the myeloid lineage including dendritic cells from lymphoid and gut tissues (5–7) and from skin (8), eosinophils in the gut mucosa (9), as well as in lymphoid CD4^+^ T cells isolated from intestinal tissue (10). Recently epidermal keratinocytes have been found to be the major IL-22BP source in the skin in steady state conditions (11). Specific to humans, this gene expresses three alternatively spliced variants called *IL22RA2v1* (IL-22BPi1), *IL22RA2v2* (IL-22BPi2), and *IL22RA2v3* (IL-22BPi3), which are co-expressed in moDCs (5, 12). The murine *Il22ra2* gene produces only one isoform, which is the homolog of human *IL22RA2v2* (13). Surface plasmon resonance (SPR) studies have been performed to estimate affinity of interaction of human IL-22BPi2 with IL-22 (14, 15). These revealed that IL-22BPi2 neutralizes the biological activity of IL-22 via formation of an exceptionally tight (Kd ≈ 1 pM) complex with IL-22 (15–18). Compared to a soluble form of the cell surface receptor sIL-22R1, the dissociation half-time (t_½_) values of the IL-22/IL-22BPi2 complex are strikingly larger (≈4.7 days for IL-22/IL-22BPi2 vs. 7 min for IL-22/sIL-22R1). Thus, IL-22BPi2 appears to exhibit a much higher affinity for IL-22 than the cell surface receptor (15). However, IL-22BPi3 displays lower affinity for IL-22 with binding kinetics similar to the IL-22/sIL-22R1 complex (15), and it is less efficient in blocking IL-22 bioactivity (12). The biological function of IL-22BPi1 that contains a 32-amino acid insertion within the reading frame at position 67 of IL-22BPi2, coded for by alternatively spliced exon-4, has not been reported, and is explored in this article.

The role of IL-22BP in disease is being elucidated, mainly through analysis of IL-22BPi2 in mouse models. Mirroring IL-22 biology, both protective and inflammatory roles have been attributed to IL-22BPi2. In a mouse model of inflammation-induced colon cancer, IL-22BPi2 produced by DC in the colon exerted a protective role by controlling tumorigenesis and epithelial cell proliferation (6). In contrast, IL-22BPi2 production is enhanced during inflammation in Crohn's Disease and ulcerative colitis and may exert pathogenic effects through blockage of protective IL-22 (9, 10). IL-22BPi2-knockout mice showed aggravation of psoriasis-induction with increased expression of IL-22-associated anti-microbial peptides, and this effect was reproduced following injection of a neutralizing antibody for IL-22BPi2 (19). IL-22BPi2 expression has also been detected in mouse dendritic cells present in the subepithelial dome of Peyer's patches where it blocks IL-22 and promotes bacterial uptake by modulating gene expression in the follicle-associated epithelium (7). rIL-22BPi2 administration to mice before sepsis induction was found to attenuate bacterial load and organ failure at the site of infection (20). The human *IL22RA2* gene locus contains single nucleotide polymorphisms (SNPs) that are associated with cancer remission (21) and risk to contract multiple sclerosis (MS) (22–25). In a mouse model for MS, experimental autoimmune encephalomyelitis (EAE), deletion of the *Il22ra2* gene conferred a less severe course of disease (26). Perriard et al. (27) demonstrated that expression of IL-22 and IL-22BP are dysregulated in MS patients. Risk SNPs acting as expression- or splicing-quantitative trait loci (e- or sQTLs) may affect overall expression levels of gene transcripts or enhance splicing of alternative transcripts, respectively (28). It remains to be investigated if and to what extent such effects are exerted by risk SNPs on *IL22RA2* transcripts.

Targeting of IL-22 may have therapeutic potential for treatment of diseases in which the IL-22 signaling pathway is implicated including inflammatory bowel disease, inflammatory skin disorders or multiple sclerosis. Currently, blocking IL-22 with anti-IL-22 monoclonal antibody is in in phase 2a trial for treatment of atopic dermatitis (29); moreover, delivery of the murine *IL22ra2* gene by cationic micelles has displayed promising potential for colon cancer therapy (30). The therapeutic potential of the two other human IL-22BP isoforms is as yet poorly understood.

In order to pave the way for a better understanding of the specific contribution of the individual IL-22BP isoforms to neuro-inflammation and MS, we characterized *IL22RA2* transcripts in myeloid cells and linked mRNA expression to secreted immunoreactive protein. We performed a biochemical characterization of the three human IL-22BP isoforms in recombinant cell systems. We identified factors involved in their folding and secretion through identification of their interactomes. We demonstrate that IL-22BPi1 is incapable of binding IL-22 in bio- and co-folding assay and is retained intracellularly through interactions with ER. We show that this retention induces a strong unfolded protein response (UPR) leading to increased protein levels of GRP78 and GRP94. In addition, we report that secretion of IL-22BPi3 is independent from the ER chaperones GRP78 and GRP94. Thus, IL-22BPi1 exerts a hitherto unknown intracellular effect unrelated to IL-22 biology.

## Materials and Methods

### Materials and Reagents

IL22RA2v2 myc-FLAG tag and IL22RA2v3-FLAG tag plasmids were, respectively purchased from Origene (RC219095) and GenScript (Ohu00490). Pfu DNA polymerase was purchased from Thermo Scientific (EP0501) and all restriction enzymes were from New England Biolabs. Taq DNA polymerase was from Thermo Fisher Scientific (10342020). Vectors expressing C-terminal Myc-FLAG-tagged IL22RA2v2, IL-2, IL-4, IL-10, IL-17, IL-22, IFN-γ, and GRP94 were from Origene. Deglycosylations were performed using Endo H (New England Biolabs, P0702s) and PNGaseF (New England Biolabs, P0704s). IFN-γ was from Peprotech (AF30002; used at 500 pg/ml), LPS from Sigma (L2630; 200 ng/ml) CpG (ODN2216; 1 μg/ml) and Poly I:C (1 μg/ml) from Invivogen. Anti-IL-22BP antibodies used throughout this work are listed in Supplementary Table 1. Further antibodies used are provided in the corresponding method sections. Predesigned TaqMan assays for *IL22RA2* (Hs00364814_m1), *IL12B* (Hs01011519_m1), *IL6* (Hs00174131_m1) and *ACTB* (Hs99999903_m1) were from Thermo Fisher Scientific. Predesigned SYBR Green primers for *GRP94* (QT00046963), *ERDj3* (QT00042560) and *GRP170* (QT00046214) were from Qiagen. Predesigned SYBR Green primers for *HPRT1* (Hs.PT.58v.45621572), *IL22RA2* (Hs.PT.58.40811), *HERP* (Hs.PT.58.21409911), *CHOP* (Hs.PT.58.3400360), *GRP78* (Hs.PT.58.22715160), *ERP44* (Hs.PT.58.4529248), and *PPIB* (Hs.PT.58.40291667) were from IDT. Additional in-house designed SYBR Green primers were from IDT and their sequences are represented in Supplementary Table 2. For silencing of *IL22RA2* we used On-TARGETplus SMARTpool human *IL22RA2* siRNA (Dharmacon; L-007974-00-0005) and non-targeting control siRNA, both used at 100 nM unless otherwise specified. Polymer-based transfection reagents Viromer BLUE (VB-01LB-00) and GREEN (VG-01LB-03) were from Lipocalyx. SubAB and a non-proteolytic mutant thereof with a S272A substitution in the A subunit (designated Mut SubAB) were purified from recombinant *E. coli* as previously described (31).

### CD14^+^ Monocyte Isolation, Maturation and Cell Culture

Buffy coats were obtained from healthy donors from the Basque Biobank (http://www.biobancovasco.org/en/) with the approval of the Ethics Committee of Clinical Investigation of the Basque Country (CEIC-E) based on provision of a detailed study protocol. Peripheral blood mononuclear cells (PBMCs) were separated through Ficoll-Paque (GE Healthcare) gradient centrifugation. Monocytes were isolated from PBMCs by CD14 positive selection using MACS CD14 microbeads (Miltentyi, 130-050-201). To differentiate isolated monocytes into immature monocyte-derived dendritic cells (moDCs), monocytes were cultured at 1 × 106 cells/ml density in Mo-DC medium, a commercially available moDC differentiation medium based on RPMI 1640 medium containing FBS, L-glutamine, IL-4 and GM-CSF (Miltenyi, 130-094-812), for 6 days, or as indicated, and an equal volume of fresh Mo-DC medium was added on day 3. Maturation of moDCs was induced by adding IFN-γ (Peprotech, AF-300-02; 500 pg/ ml) for overnight priming (16 h) followed by late addition of LPS for 6 hrs (Sigma, L6143; 2 μg/ml) to the culture or, alternatively by addition of CpG (Miltenyi, 130-100-243; 1 μg/ml) or LPS/Poly(I:C) (Sigma, P1530; 1 μg/ml) for 6 h. HEK293, HeLa and A549 were cultured in DMEM supplemented with 10% FBS and 2 mM L-glutamine; U937 cells were cultured in RPMI supplemented with 0.05 mM β-mercaptoethanol, 10 % FBS and 2 mM L-glutamine.

### FACS of Monocytes and Flow Cytometry of moDCs

PBMCs were obtained from freshly isolated buffy coats of healthy donors using Ficoll-Hypaque gradient (ThermoFisher). Monocytes were isolated using Pan Monocyte Isolation kit (Miltenyi) following manufacturer's instructions. Monocytes were labeled for 15 min in dark conditions with PE conjugated anti-human CD14 (Miltenyi, 130-110-519) and FITC conjugated anti-human CD16 (Miltenyi, 130-106-703) in sorting buffer (HBSS, FBS 2%; EDTA 2 mM, HEPES 25 mM) prior to cell sorting. Monocyte subpopulations were sorted in a FACSJazz (BD) cell sorter following the strategy described in Supplementary Figure 1. For assessing maturation state, moDCs were labeled with Mo-DC Differentiation Inspector (Miltenyi, 130-093-567), a commercially available mouse monoclonal conjugated antibody cocktail containing CD14 antibody conjugated to fluorescein-isothiocyanate (FITC; clone Tük4, isotype: mouse IgG2a), CD83 conjugated to allophycocyanin (APC; clone: HB15, isotype: mouse IgG1), and CD209 conjugated toR-phycoerythrin (PE; clone DCN-47.5.4, isotype: mouse IgG1) or isotype control cocktail containing monoclonal mouse IgG2a-FITC, IgG1-PE, and IgG1-APC conjugates in corresponding concentrations (Miltenyi, 130-093-567) following manufacturer's instructions. Gating strategy consisted of a size-complexity discrimination of moDCs and selection of single cells prior to label discrimination. CD14 (FITC) and CD209 (PE; which corresponds to DC-SIGN) were used to select moDCs (CD14^−^CD209^+^). CD209 (PE) and CD83 (APC) were used to discriminate between immature (CD209^+^CD83^−^) and mature (CD209^+^CD83^+^) cells. Raw CD209/CD83 flow cytometry data of immature and mature moDCs are provided in Supplementary Figure 2. Raw CD14/CD209/CD83 flow cytometry data of immature and IFN-γ/LPS-matured CD16^−^CD14^+^ and CD16^+^ monocyte-derived DCs and corresponding cytokine measurements by qPCR are available in Supplementary Data 1.

### RT-PCR, qPCR and Conventional PCR

#### Reverse Transcriptase PCR(RT-PCR)

RNA was extracted with TRI Reagent (Sigma, T9424) following manufacture's protocol, and quantified using Nanodrop 2000c spectrophotometer (Thermo Scientific). Five hundred Nano gram of RNA were reverse-transcribed to cDNA according to manufacturer's instructions (Thermo Scientific, 4368814) in a volume of 20 μl on a Veriti thermocycler (Applied Biosystems).

#### Quantitative PCR (qPCR)

One microliter of cDNA was amplified by qPCR using primers and SYBR Green (Thermo Scientific, 4385616) or Taqman probes on a 7500 Fast Real Time PCR System (Applied Biosystems). The primers used in each figure are indicated in Supplementary Table 2. qPCR with amplification program for SYBR Green was: one cycle at 95°C for 20 s, followed by 40 cycles at 95°C for 3 s and 60°C for 30 s, and concluding with a dissociation stage. qPCR with amplification program for Taqman was: one cycle at 50°C for 2 min, followed by one cycle at 95°C for 10 min. This was followed by 40 cycles at 95°C for 15 s and 60°C for 1 min and concluded with a dissociation stage. The threshold for detection was set within the linear phase of the logarithmic amplification plot. All targets were measured in technical duplicates or triplicates. Quality negative controls were included in all runs, and melting temperatures were analyzed for SYBR Green qPCRs; as well, qPCR products sizes were analyzed by gel electrophoresis. Levels of expression of each gene were normalized to the housekeeping genes used, as indicated, and expressed as 2^−ΔΔ*Ct*^ relative to the average of the control group.

#### Conventional PCR

Conventional PCR was carried out on a Veriti thermocycler (Applied biosystems). All PCR reactions were performed using *Taq* DNA polymerase (Thermo Fisher Scientific, 10342020) in the presence of dNTP's (Thermo Fisher Scientific, 10297018). Ten microliter of each cDNA product were amplified with the following amplification program: initiation step, 94°C for 45 s, followed by 35 cycles of [denaturation step at 94°C for 45 s; elongation step in which annealing temperatures were adjusted to the melting temperatures of each pair of primers, and elongation step at 72°C for 10 s]. A final extension step was performed at 72°C for 10 min. Products were run on 2% agarose (w/v) gels (Agarose: Sigma, A9539) in Tris acetate-EDTA buffer with SYBR Safe. Gels were visualized with the ChemiDoc Imaging System (Bio-rad).

### Cloning Procedures

#### Cloning of IL22RA2v1 Into pCMV6-Entry Vector

The cDNA coding for IL-22RA2v1 (isoform-1) was initially amplified with *Pfu* polymerase from two overlapping fragments with primers including terminal *MluI* and *SgfI* restriction sites starting from retrotranscribed RNA extracted from immature moDCs. PCR products were digested with *MluI* and *SgfI* restriction enzymes and cloned into pCMV6-Entry vector.

#### Subcloning of IL22RA2v2 Into TET-Express System

The multiple cloning site of pTRE3G-BI-mCherry vector (Clontech, 631333) was modified by including the following sequence by oligo overlapping: BamHI-MluI-BspEI-FLAG-Stop-Stop-EaglI. The IL22RA2v2 sequence was amplified from the original PCMV6-Entry vector (Origene RC219095) with *Pfu* polymerase, digested with *BglII* and *MluI*, and cloned into the modified pTRE3G vector.

#### Cloning IL22RA2v1 Alternatively Spliced Exon Into IL-2

Exon-4 in IL-22RA1-containing pCMV6-entry vector was amplified with FW IL-2 and RV IL-2 cloning primers using *Pfu* polymerase. Both the purified fragment of 124 bp and IL-2 vector were digested using *XbaI* and purified. To avoid religation, the vector was dephosporylated with shrimp alkaline phospatase (SAP; Promega M820A; 15 min 37° and 15 min at 65°). Ligation with T4 DNA Ligase (New England BioLabs; M0202S) and transformation of Mach 1-competent cells (Thermo; C862003) were performed. All constructs were verified to be error free by sequencing on both strands.

### Transient Transfection, Protein Extraction and Cell Manipulations

Transient transfections of HEK293 or HeLa cells were conducted at 60–70% confluency using MACSfectin Reagent (130-098-412, Miltenyi Biotec). For TETexpress-inducible system, transactivator was added 24 h after transfection following manufacture instructions (Clontech). At indicated times, medium was removed and stored at −80°C in presence of protease inhibitors (Roche, 11697498001); cells were collected and washed three times with PBS before addition of lysis buffer (300 mM NaCl, 50 Mm NaH_2_PO_4_ pH 8, 1% Triton X-100) or RIPA buffer (25 mM Tris/HCl 150 mM NaCl, pH 7.6, 1% NP - 40, 1% sodium deoxycholate, 0.1% SDS) as indicated, both containing protease inhibitors cocktail. Cell lysis was done for 30 min on ice followed by centrifugation at 21.000 × g at 4°C. Protein fraction was collected from the supernatants and immediately processed for immunoprecipitation or stored at −80°C for further analysis. Protein concentrations were determined using Bradford (Bio-Rad, 5000006) or BCA (Bio-Rad, 23225) colorimetric assays. Absorbance was measured with Varioskan Flash (Thermo Fisher Scientific). Conditioned media was also collected and proteasome inhibitors cocktail added. For acetone precipitations, 4 h before collection, conditioned medium was removed and cells were carefully washed three times with pre-warmed serum-free medium (Lonza, 12–764Q) supplemented with 2 mM L-glutamine. Serum-free medium (SFM) was added and left for that period in culture conditions. Four volumes of ice-cold acetone were added to the medium, mixed and incubated for 15 min on ice. Samples were centrifuged at 12,000 × g for 10 min at 4°C. Supernatants were carefully removed and precipitates were resuspended in loading buffer or in water for enzymatic treatment. Proteasome inhibition: Protein degradation was studied as published (32). Briefly, 24 h after transfection a mixture of proteasome inhibitors (5 μM lactacystin, 5 μM MG132, and 1 mM epoxomycin) was added and 18 hrs later, cells were harvested, lysed and analyzed by immunoblotting. Cell fractionation: Cell fractionation was carried out according to Holden and Horton protocol (33).

### Immuno-/Affinity Purification

Three different purification resins were used depending on the protein of interest. Anti-FLAG resin (GenScript, L00432), anti-c-Myc resin (Sigma, E6654) and S-protein agarose (Millipore, 69704) were used following manufacturers' instructions. All purifications were performed o/n at 4°C on a rotating wheel. Samples were washed six times with equilibration buffer (50 mM Tris, 150 mM NaCl, pH 7.4, 0.1% Tween 20). Elution buffer (0.1 M glycine, 0.5% SDS, 0.15 M NaCl pH 2) was then added to the resin, samples were incubated 5 min at RT with gentle mixing, resins were centrifuged at 300 × g for 2 min, supernatants were carefully collected avoiding the resin absorption and pH was adjusted adding 1:10 of 1 M Tris pH 10.

### IL-22BP Bioassay

IL22BPi1, IL22BPi2 and IL-22 were independently transfected into HEK293 as previously mentioned. After 24 h, secreted fractions of IL22BPi1 and IL22BPi2 were quantified by ELISA (see below). Different dilutions and exposure times of IL-22 were assessed to A549 to stablish the optimal time and dose-response of STAT3 phosphorylation, which was measured by western blot. Equal amounts of IL22BPi1 or IL22BPi2 were mixed with the selected volume of IL-22 and filled to the same final volume with conditioned media. Mixture was incubated at 37°C for 1 h prior to addition to A549. After the exposure time, the A549 protein fraction was extracted and analyzed by immunoblot.

### IL-22BP Isoform Interactome Analysis

Protein interactomes of IL-22BP isoforms were purified by anti-FLAG affinity chromatography as described above, and eluted fractions were resolved on 10% precast SDS-PAGE gels (Bio-Rad, 4561034). Gels were silver stained following manufacture's protocol (Thermo Fisher Scientific, 24612), washed, and gel bands were sliced. Each band was digested with trypsin following a standard in-gel digestion protocol based on Shevchenko et al. (34) with minor modifications (34). The resulting peptides were resuspended in 0.1% formic acid, separated using online NanoLC and analyzed using electrospray tandem mass spectrometry. Peptide separation was performed on a nanoACQUITY UPLC system connected to a SYNAPT G2-Si spectrometer (Waters, Milford, MA, USA). Samples were loaded onto a Symmetry 300 C18 UPLC Trap column of 5, 180 μm × 20 mm (Waters, Milford, MA, USA), connected to a BEH130 C18 column of 1.7, 75 μm × 200 mm (Waters, Milford, MA, USA). The column was equilibrated in 3% acetonitrile and 0.1% FA. Peptides were eluted at 300 nL min^−1^ using a 60-min linear gradient of 3–50% acetonitrile. A SYNAPT G2-Si ESI Q-Mobility-TOF spectrometer (Waters, Milford, MA, USA) equipped with an ion mobility chamber (T-Wave-IMS) for high definition data acquisition analyses was used for the analysis of the peptides. All analyses were performed using electrospray ionization in a positive ion mode. Data were post-acquisition lock-mass corrected using the double-charged monoisotopic ion of [Glu^1^]-fibrinopeptide B. Accurate LC-MS data were collected in HDDA mode, which enhances signal intensities using the ion mobility separation. Mascot searching engine (MatrixScience) against Uniprot/Swissprot human database was performed. Searching parameters were delimited as follows: Peptide mass tolerance of 10 ppm and 0.2-Da fragment mass tolerance were. Carbamidomethylation of cysteines was selected as the fixed modification and oxidation of methionine as a variable modification for tryptic peptides. Proteins identified with at least two peptides with an FDR < 1% were kept for further examination.

### Immunoblotting

Equal quantities of cell lysates, volumes of eluted fractions, or conditioned media were loaded on SDS-PAGE gels (10 or 12%) and transferred to PVDF membranes for immunoblotting; blots were blocked with 2% casein o/n at 4°C and subsequently incubated with primary antibodies o/n at 4°C. Blots were washed with TBST buffer 3 times for 10 min each. HRP-conjugated secondary antibodies were incubated for 1 h. Immunoreactivity was assessed using a chemiluminescence substrate (Bio-Rad, 1705061) and measured using a ChemiDoc Imaging System (Bio-rad). Densitometry values were normalized to a control loading protein or to the Ponceau staining) using Image Lab Software (Bio-Rad). Primary antibodies were anti-FLAG (Rb, Proteintech; 20543-1-AP; 1:1000, & ms, Sigma; F1804), anti-GRP94 (Rt, Enzo; ADI-SPA-850; 1:1000), anti-IL-22BP (Gt, R&D Systems; AF1087 and BAF1087; 1:1000 each, and Ms, Proteintech; 66190; 1:1000), anti-pSTAT3 (Rb, R&D Systems; AF4607; 1:1000), anti-actin (Rb, Sigma-Aldrich; A2066; 1:100), anti-tubulin (Ms, GenScript; A01490; 1:1000), anti-GAPDH (Ms, Millipore; ABS16; 1:2000), anti-GRP78 (Gt, R&D Systems; AF4846; 1:1000), anti-GRP170 (Ms, IBL; 10301; 1:100), anti-CNX (Rb, Enzo; ADI-SPA-860; 1:1000), anti-ERdj3 (Rb, Proteintech; 15484-1-AP; 1:1000), anti-KDEL (Ms, Enzo; ADI-SPA-827; 1:1000), anti-histone H3 (Rb, Cell Signaling; 4499; 1:2000), anti-PPIB (Rb, Abcam; 16045; 1:500). All HRP-conjugated secondary antibodies were purchased from Jackson immunoResearch.

### IL22BP ELISA

An in-house ELISA was developed for sensitive IL-22BP detection. High binding ELISA plates (Sarstedt, 82.1581.200) were coated with 100 μl of 0.5 μg/ml antigen-purified goat polyclonal IL-22BP antibody (R&D systems, AF1087; immunogen used was recombinant human IL-22BP Thr22-Pro231 coinciding with mature IL-22BPi2) in coating solution (50 mM Tris/HCl, 150 mM NaCl, pH 8,5) and incubated o/n at 4°C. The next day, coating mix was removed followed by three washings steps with 200 μl of washing solution (50 mM Tris/HCl, 150 mM NaCl, 0.05% Tween 20, pH 7,4) each; 200 μl of blocking solution (50 mM Tris/HCl, 150 mM NaCl, 0.1% casein, pH 7,4) was added to the wells and incubated o/n at 4°C. Blocking solution was removed followed by three washings with 200 μl of washing solution each. Semilog dilutions of biosamples were added to the plate (100 μl each) prediluted 1:10 in assay buffer (50 mM Tris/HCl, 150 mM NaCl, 0.1% casein, 0.05% Tween 20, pH 7,4), with recombinant IL-22BP human protein-His tag (Sino biologicals, 11025-H08H) used as standard. Samples and standard were incubated 2 h at 37°C. Samples were removed and wells were washed six times with 200 μl of washing solution each. Antigen-purified goat polyclonal IL-22BP biotinylated antibody (R&D Systems, BAF1087) was added to each well in assay buffer at 0.5 μg/ml dilution and incubated for 1 h at 37°C followed by six washings. Biotinylated detection was performed with streptavidin conjugated to horseradish peroxidase (R&D systems, DY998) diluted in assay buffer 1:200 for 1-h incubation time at 37°C. Streptavidin-HRP mixture was removed and plates were washed six times. ELISA was developed with 95 μl of TMB substrate solution (Thermo Scientific, 34028) in a 30 min incubation period. Reaction was stopped with 95 μl of 2 M H_2_SO_4_ and absorbance was read at 450 nm using Varioskan Flash (Thermo Scientific). Preliminary experiments demonstrated that the AF1087 or BAF1087 antibodies detected each of the three IL-22BP isoforms in western blot in the conditioned media of individually transfected cells.

### Immunofluorescence Microscopy

Cells in coverslips were fixed with cold 4% paraformaldehyde for 10 min and washed 3 times with PBS. Microscopy buffer containing 0.2% Triton X-100 and 3% BSA in PBS, pH 7.4 was used in all subsequent steps. Permeabilization was performed and non-specific staining was blocked with microscopy buffer for 30 min. Primary antibodies were incubated for 1 h at RT in microscopy buffer. Then cells were washed 3 times for 10 min with PBS. Secondary antibodies were incubated in dark conditions for 1 h at RT in microscopy buffer. All the downstream steps were also performed in dark conditions. Following secondary incubations, coverslips were incubated with PBS containing DAPI for 5 min at RT followed by two washes with PBS for 10 min. Finally, coverslips were mounted on glass slides using Fluoromont (Southern Biotech 0100-01). Images were acquired in a Leica TCS STED CW SP8 super-resolution microscope. For confocal immmuofluorescence microcroscopy, we used the following antibodies: anti-IL22RA2 (R&D; AF1087 and BAF1087, 1:20 each), anti-IL22RA2 (Proteintech; 66190-1-Ig), anti-TGOLN (Atlas; HPA012723-100ul, 1:500), anti-ERp72 (Enzo; ADI-SPS-720-F; 1:500), donkey anti-goat A647 (Jackson ImmunoResearch; 705-605-147; 1:500), Streptavidin DyLight 488 (Tebu-bio; 039S000-41; 1:4000), donkey anti-rabbit A647 (Jackson Immunoresearch; 711-605-152; 1:500), as well as DAPI (Sigma; 10 μg/ml).

### Statistical Analysis

Statistical analysis was performed for quantitative assays with the GraphPad Prism software, specific tests are included in the legends to figures.

## Results

### IL-22BP Protein Levels Reflect *IL22RA2* mRNA Levels Produced by Monocyte-Derived Dendritic Cells

**Figure 1:**
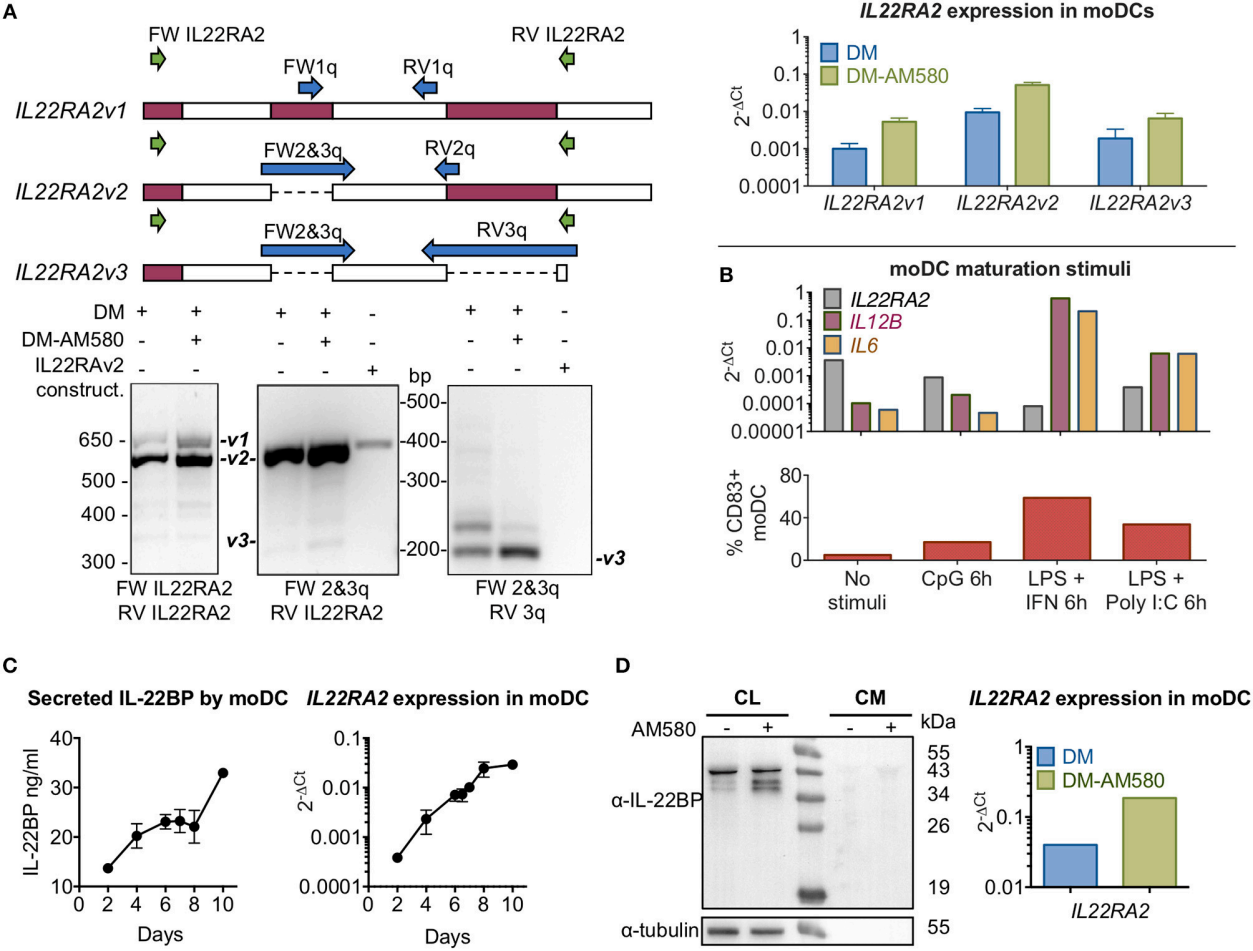
IL-22BP protein and mRNA levels in monocyte-derived DCs. **(A)** (Upper left) Diagram showing exons with the primer-binding positions for *IL22RA2* variant discrimination strategy. (Lower left) Expression of *IL22RA2v1, v2*, and *v3* in moDC after 6 days of culture in differentiation medium (DM) ± AM580, a retinoic acid receptor agonist, by RT-PCR. IL22RA2v2 expression vector was used as positive control for *IL22RA2v2* expression. (Upper right) Expression of *IL22RA2* variants in moDC following 6 days of culture in DM ± AM580 by RT-qPCR using the isoform-specific primers indicated in the left diagram (mean ± SEM; *n* = 3) relative to the housekeeping gene *GAPDH*. **(B)** Effect of different maturation stimuli (CpG and LPS + IFN-γ or Poly I:C) on *IL22RA2, IL12B*, and *IL6* expression in moDC after 6 days of culture in DM by RT-qPCR using Taqman probes relative to the housekeeping gene *ACTB*. Extent of maturation is represented as percentage of CD83^+^ fluorescence by flow cytometry. Data showing representative experiment of 3 performed. **(C)** IL-22BP secretion by moDC cultured in DM detected by ELISA corresponds to *IL22RA2* mRNA expression levels analyzed by RT-qPCR using Taqman probes and both increase over the cultivation period (mean ± SEM, *n* = 2). **(D)** Cell lysates (CL) of moDCs and conditioned media (CM) were harvested after 6 days in DM ± AM580 and immunoblotted for detection of IL-22BP (IL-22BP antibody 1) using tubulin as a loading control, *IL22RA2* mRNA expression relative to the mean of the housekeeping genes *GAPDH* and *ACTB* was measured by RT-qPCR using pre-designed SYBR Green primers from the same cells. Representative experiment out of three performed. All primers used are listed in Supplementary Table 2.

The three *IL22RA2* mRNA variants were detected in immature moDCs (primary monocytes cultivated 6 days in differentiation medium (DM) containing granulocyte-macrophage colony-stimulating factor (GM-CSF) and IL-4) by RT-PCR using both best coverage (BC; i.e., primers designed to detect the three isoforms by annealing to shared regions in the expressed products) and isoform-specific designed primers, and products were verified via agarose gel electrophoresis (Figure 1A). Expression levels of the three transcripts increased in the presence of AM580, a retinoic acid receptor agonist, in agreement with Martin et al. (5) (Figure 1A). Maturation of moDCs with CpG, IFN-gamma + LPS or polyI:C + LPS decreased *IL22RA2* gene expression (Figure 1B), with the extent of the decrease proportional to that of the degree of maturation (Supplementary Figure 2). Given that the three isoforms share the same functional signal peptide and should therefore be secreted, and that secretion of IL-22BP by natural producer cells has not been reported in the literature, we set out to demonstrate IL22BP immunoreactivity in both cell lysates and the culture medium by means of an in-house developed antigen-purified goat polyclonal ELISA (see section Materials and Methods) capable of detecting 30 pg/ml recombinant IL22BP. In immature moDCs cells, strongly increasing levels of *IL22RA2* mRNA were observed by qPCR over a 10-day cultivation period (Figure 1C), but corresponding IL-22BP levels in the culture medium of these moDCs as measured by ELISA increased only modestly (Figure 1C). In western blot, intracellular IL-22BP immunoreactive protein bands were detected with molecular masses extending from around 38 to 40 kDa that were increased in AM580-treated moDCs (Figure 1D). However, individual secreted IL-22BP isoforms could not be resolved in culture medium of moDCs (Figure 1D) or following concentration of medium with acetone (Supplementary Figure 3), and this may be due to the low levels of secreted IL-22BP as detected by ELISA. Additional experiments to detect IL-22BP in the monocytic U937 cell line [reported to produce *IL22RA2* transcripts in (13)] or moDC by western blot using distinct anti-IL-22BP antibodies are presented in Supplementary Figure 3. Therefore, we used recombinant expression vectors transfected into HEK293 cells to gain more insight into the mechanisms governing secretion of IL-22BPi1, 2 and 3. Vectors expressing IL-22BPi2 and -BPi3 were commercially available, an expression vector for IL-22BPi1 was constructed from moDC mRNA as described in section Materials and Methods.

### IL-22BPi1 Is Poorly Secreted, Retained in the ER and Degraded by the Proteasome

**Figure 2:**
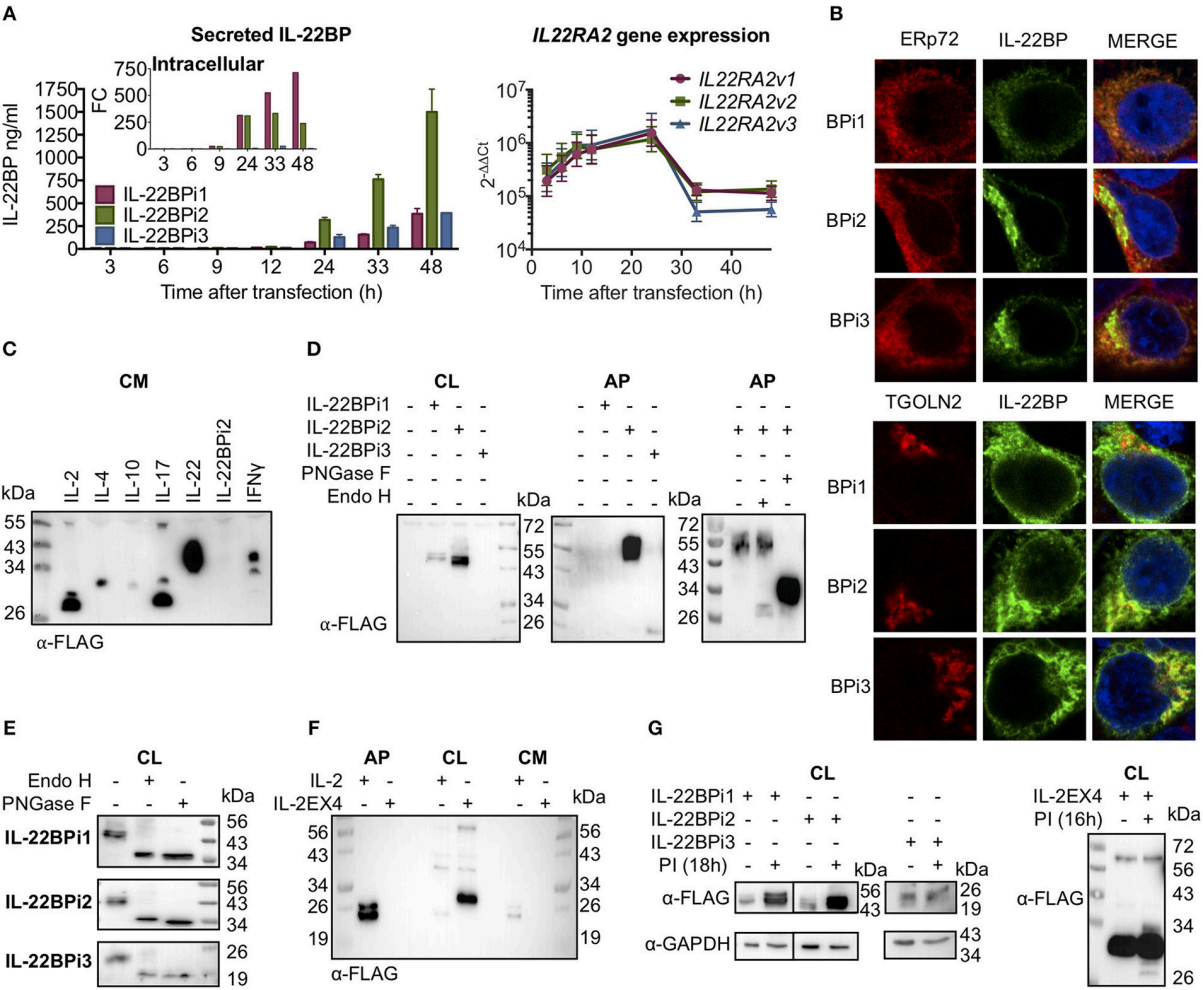
Differential secretion, intracellular retention and degradation by the proteasome of IL-22BP isoforms. Data refer to HEK293 cells transiently transfected for 24 h unless otherwise specified. **(A)** HEK293 cells were transiently transfected with expression vectors encoding IL-22BPi1, BPi2, or BPi3. Intracellular and secreted IL-22BP protein levels were measured by WB (FLAG Ab) and ELISA, respectively. Transfection efficiency was measured by *IL22RA2* RT-qPCR relative to the housekeeping gene *GAPDH* (mean ± SEM; *n* = 3). **(B)** Confocal microscopy of IL-22BP isoforms (green, IL-22BP Ab 4 with either ERp72 or trans-Golgi marker TGOLN2 (red) in transfected HEK293 cells; cells were counterstained with DAPI. **(C)** Secreted recombinant IL-22BPi2 is not detected by WB in unconcentrated conditioned media (CM). Various cytokine expression vectors, as indicated, were transiently transfected and CM were collected and immunoblotted for FLAG. **(D)** Detection of IL-22BP isoforms in cell lysates (CL) and acetone-precipitated CM (AP) by FLAG Ab. Secreted IL-22BPi2 was treated with PNGase F or Endo H. **(E)** Intracelullar isoforms of IL-22BP were treated with PNGase F or Endo H and detected by FLAG Ab. **(F)** HEK293 cells were transfected for 24 h with expression vectors for either IL-2 or IL-2EX4. The latter was generated by subcloning exon-4 from IL22RA2v1 into the open reading frame of IL-2 through its unique *Xba1* position. Detection of resulting proteins in AP, CL, CM by FLAG Ab. **(G)** HEK293 cells were transiently transfected with the same expression plasmids as in **(A)** or IL-2EX4; 24 h later, cells were treated with a mix of proteasome inhibitors (PI) (5 μM lactacystin, 5 μM MG132, and 1 mM epoxomicin), and 18 h later cells were lysed and immunoblotted for FLAG using GAPDH as a loading control. All data are from at least 3 independent experiments.

C-terminally myc-FLAG-tagged IL-22BPi1, IL-22BPi2 and IL-22BPi3 expression plasmids were individually transfected into HEK293 cells, and secreted IL-22BP levels were subsequently measured by ELISA. While high levels of IL-22BPi2 were observed in the culture medium (Figure 2A), those of both IL-22BPi1 and IL-22BPi3 were much lower, even if transfection efficiencies as measured by RT-qPCR through quantification of mRNA levels were similar (Figure 2A). Conversely, intracellular levels of IL-22BPi1, but not of the other two isoforms, increased over time after transfection (Figure 2A, *inset*) suggesting that the former's poor secretion was related to preferential intracellular accumulation. In immunofluorescence microscopy, IL-22BPi1 formed a perinuclear diffuse granular staining pattern tending to co-localize with the endoplasmic reticulum (ER) marker ERp72 but not with the trans-Golgi marker TGOLN2 (Figure 2B). IL-22BPi2 and IL-22BPi3, in contrast, were mainly visible as densely staining deposits located on one side outside the nucleus that co-localized with TGOLN2 but less so with ERp72. These data imply efficient export of IL-22BPi2 and IL-22BPi3 from ER to the Golgi apparatus, but not of IL-22BPi1 that seems to be retained predominantly within the ER.

Next, we compared the detection of secreted IL-22BPi2 with a series of cytokines that were, like IL-22BPi2, expressed with C-terminal myc-FLAG tags. In contrast to these cytokines, IL-22BPi2 was not detected in the cell culture medium by western blotting using an anti-FLAG antibody (Figure 2C). However, secreted IL-22BPi2 and IL-22BPi3 were detected with anti-FLAG after concentration of culture medium with acetone (AP), while IL-22BPi1 was not (Figure 2D). Treatment of cell lysates with peptide *N*-glycosidase F (PNGase F, which cleaves all *N*-linked glycans) or endoglycosidase H (Endo H, which cleaves within the chitobiose core of high-mannose but not complex glycans), revealed that the three intracellular isoforms contain high-mannose-type *N*-glycans compatible with 3 predicted *N*-glycosylation sites for IL-22BPi1 and IL-22BPi2, and 1 for IL-22BPi3 (NetNGlyc 1.0 server; Figure 2E). Compared to the cell-associated protein, secreted IL-22BPi2 gained around 8 kDa in Mr (56 vs. 48 kDa) and was resistant to Endo H, but not PNGase F, indicating the sole presence of complex *N*-glycans and thus transit via Golgi (Figure 2D, rightmost panel). We also found that the secreted deglycosylated protein was more sensitive to detection in western blot than the glycosylated one (Figure 2D). In order to determine whether poor secretion of IL-22BPi1 is due to the presence of an extra 32-amino acid stretch coded by the alternatively spliced exon that is absent in IL-22BPi2 and IL-22BPi3, we cloned this sequence into IL-2, an efficiently secreted protein (Figure 2C). We inserted the sequence into the unique *XbaI* site of IL-2 (position 233 following ATG initiation codon), maintaining the integrity of the original reading frame after the insertion as is the case in IL-22BPi1. IL-2, but not IL-2EX4, was detected in acetone precipitates of culture medium while the latter was enriched in cell lysates (Figure 2F). Biochemical fractionation of transfected cells to generate separate cytosolic, membranous organelles (MO), nuclear and insoluble fractions followed by immunoblot, revealed that IL-2EX4 similar to the three IL-22BP isoforms, was detectable in the MO fraction, and that none of these proteins sedimented in the insoluble fraction as aggregates (Supplementary Figure 4).

We asked whether the poor secretion of IL-22BPi1 and IL-2EX4 could be due to high intrinsic tendency to misfold. Misfolded proteins are known to be intercepted by the endoplasmic reticulum quality control system (ERQC) and delivered for ER-associated degradation (ERAD) by the proteasome (35). Thus, increased intracellular protein levels upon pharmacological inhibition of the proteasome point to misfolding reactions taking place. Figure 2G shows increased levels of IL-22BPi1 and IL-2EX4 and, noteworthy, also of IL-22BPi2 upon treatment of transfected cells with proteasome inhibitors, while IL-22BPi3 appeared not to be affected.

### IL-22 “Chaperones” the Secretion of IL-22BPi2 and IL-22BPi3 but Not of IL-22BPi1

**Figure 3:**
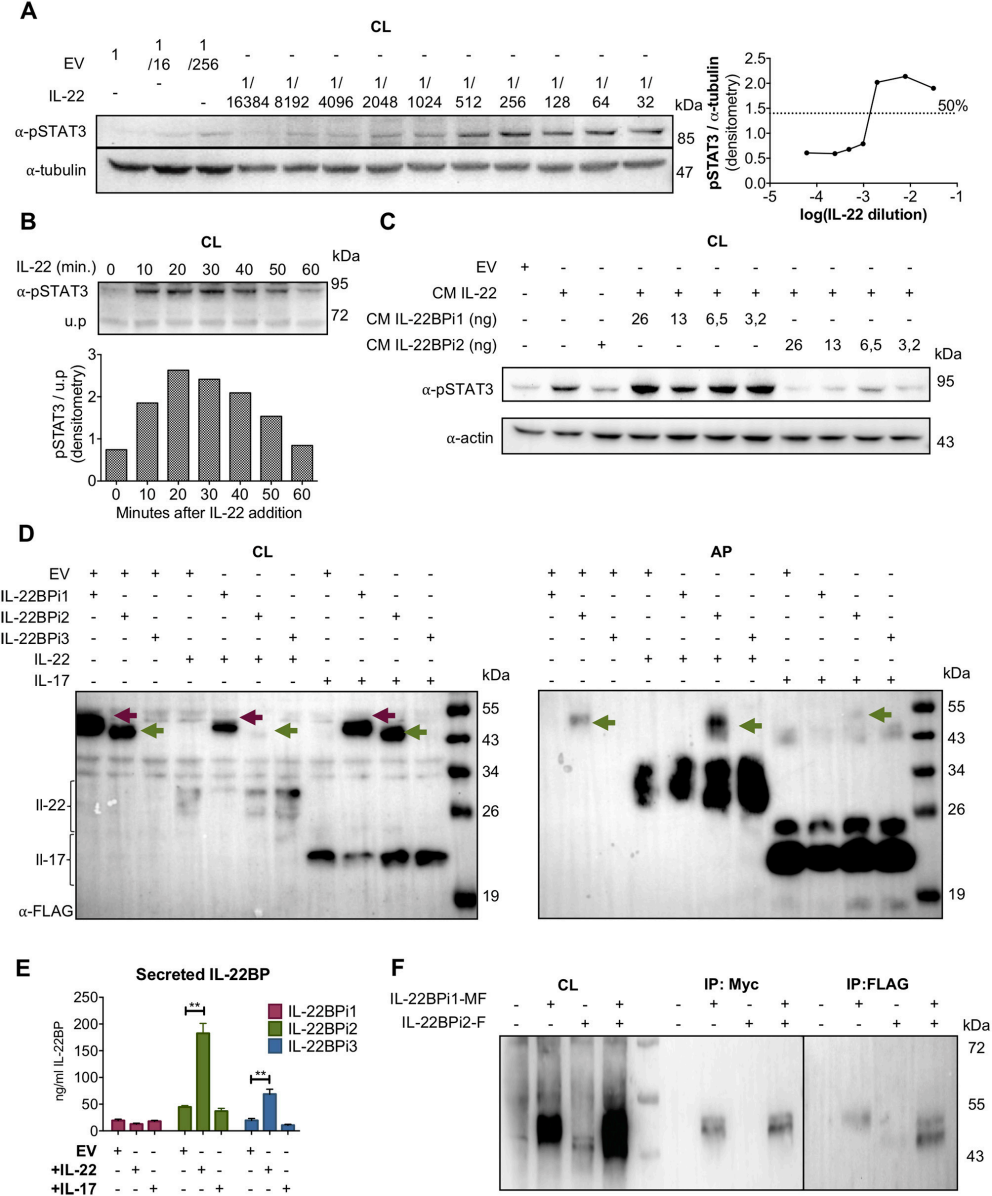
IL-22BPi1 does not interact with IL-22 or IL-22BPi2. **(A)** A549 cells were exposed for 30 minutes to dilutions of IL-22-containing culture medium (CM) previously produced by *IL22*-transfected HEK293 cells. A549 cell lysates (CL) were immunoblotted for pSTAT3 and tubulin as loading control. The relative densitometry of pSTAT3 normalized to that of tubulin is also represented. **(B)** A549 cells were treated with the optimum IL-22 dilution from A (1/512) for different periods of time, lysed and immunoblotted for pSTAT3. An unspecific protein band (u.p.) was used as loading control. **(C)** IL-22BP concentration in conditioned medium (CM) of transfected HEK293 cells was measured by ELISA, and the indicated amounts in nanograms (ng) of IL-22BPi1 or IL-22BPi2 were pre-incubated for 1 h at 37°C with the selected IL-22 concentration from **(A)**. A549 were exposed to the pre-incubated combinations for 20 min. An excess of IL-22BPi2 was used as phosphorylation blocking control (lane 3). Cells were lysed and immunoblotted for pSTAT3 and actin as loading control. **(D)** HeLa cells were co-transfected with the indicated expression plasmids, 24 h later cells were lysed and the CM was subjected to acetone precipitation (AP), and proteins were immunoblotted for FLAG. Intracellular IL-22BPi1 is indicated with dark purple arrows, intracellular and secreted IL-22BPi2 is indicated with green arrows, and co-expressed IL-22 and IL-17 are also indicated. **(E)** Conditioned media (CM) from 3 independent experiments, in which expression vectors for the three IL-22BP isoforms were individually transfected into HEK293 cells together with either IL-17 or IL-22 expression vectors or an empty vector control (EV), were analyzed 24 h after transfection by ELISA for IL-22BP (mean ± SEM; *n* = 3; ^**^p < 0.01 by unpaired *t*-test). **(F)** IL-22BPi1 does not interact with IL-22BPi2. IL-22BPi1-MF expression plasmid containing Myc and FLAG tags was co-transfected with an inducible pTRE3G-based vector expressing IL-22BPi2 with only a FLAG tag. After 24 h, cells were induced for IL-22BPi2 production by adding Tet-Express activator to the medium for a further 24 h. Cells were lysed and immunoprecipitated with anti-Myc agarose, the flow-through fractions were then further subjected to FLAG immunoprecipitation. CL and eluted fractions were immunoblotted for FLAG.

We tested the capacity of IL-22BPi1 to interact with IL-22 in both a bioassay and a co-folding assay. The bioassay was based on inhibition by IL-22BP of IL-22-induced STAT3 phosphorylation in the A549 cancer cell line. First, we determined both the largest dilution of conditioned medium (CM) from HEK293 transfected with an IL-22 expression vector still able to induce maximal phosphorylation of STAT3 in A549 cells, and the time point after addition of IL-22 at which STAT3 phosphorylation is most pronounced (i.e., dilution 1/512 and 20 min, respectively; Figures 3A,B). Next, we assayed different concentrations of IL-22BPi1 or BPi2 using CM of transfected HEK293 cells in which IL-22BPi1 or IL-22BPi2 were determined by ELISA. Inhibition of STAT3 phosphorylation by IL-22BPi2 was consistently observed, while identical quantities of IL-22BPi1 did not impair IL-22 mediated-STAT3 phosphorylation (Figure 3C). Under the conditions of this assay, IL-22BPi1 does not seem to neutralize the biological activity of IL-22.

For implementation of the co-folding assay we found inspiration in earlier work done on IL-15 and IL-15Rα. In dendritic cells, the α-chain of the IL-15 receptor acts as a chaperone to stabilize IL-15 within the secretory tract against proteasome degradation and to export it in a complex to the membrane in its bioactive conformation (32, 36). IL-22BP can be produced by a CD4^+^ subset of tissue-infiltrating T lymphocytes (10), and given that various subsets of lymphocytic cells (T and ILC) are known producers of IL-22 (1, 2), the theoretical possibility exists that IL-22BP and IL-22 may occur co-expressed in specific, yet to determine natural circumstances. We thus asked if in the foldase- and chaperone-rich, folding-permissive environment of the ER, a cytokine-receptor co-assembly interaction may take place between IL-22 and IL-22BPi1 that would rescue misfolding of the latter and augments its secretion. As demonstrated for IL-15/IL-15Rα these interactions can be reproduced in transfected human cell lines (32). We co-transfected the three isoforms of IL-22BP each with IL-22, or as negative control IL-17, a cytokine reported not to bind any IL-22BP isoform (12). Interestingly, co-expression of IL-22BPi2 and IL-22 strongly decreased intracellular and increased secreted level of the former in western blot, suggestive for productive interactions taking place enhancing folding and/or transit of IL-22BPi2 (Figure 3D). However, increased secretion in the presence of IL-22 was not seen for IL-22BPi1. In ELISA, co-expression of IL-22 significantly enhanced secretion of IL-22BPi2 and IL-22BPi3 but not that of IL-22BPi1 (Figure 3E). Co-expression of IL-17 did not have any effect on IL-22BP isoform levels (Figures 3D,E). In light of these findings, the extra 32-amino acid sequence in IL-22BPi1 appears to have disrupted this isoform's capacity for interaction with IL-22. Given the predominant ER localization of IL-22BPi1, its inability to bind to IL-22 as well as its co-production with IL-22BPi2 in natural producer cells (Figure 1), we asked whether IL-22BPi1 had the capacity to physically interact with IL-22BPi2. We co-expressed myc-FLAG-tagged IL-22BPi1 with FLAG-tag-only IL-22BPi2 in HEK293 cells. Purification with anti-myc resin only recovered IL-22BPi1 while further purification via anti-FLAG resin recovered IL-22BPi2 from the flow-through fraction, suggesting that both isoforms do not interact with each other (Figure 3F).

### IL-22BPi1 and IL-22BPi2, but Not IL-22BPi3, Bind the Substrate-Binding Domain (SBD) of GRP78

**Figure 4:**
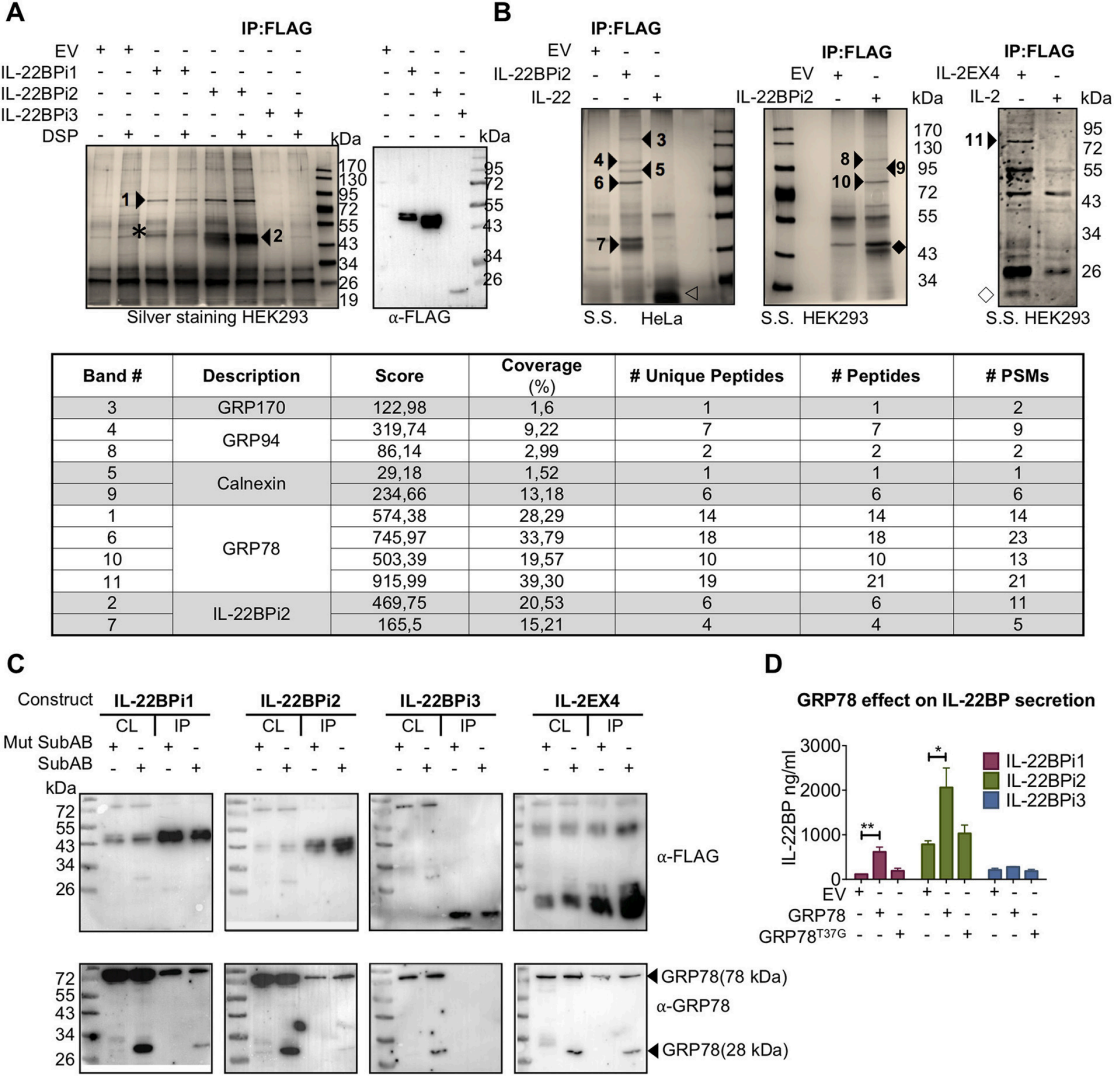
IL-22BPi1 and IL-22BPi2, but not IL-22BPi3, are *bona fide* client proteins of GRP78. (**A,B)** Identification of GRP78, GRP94, GRP170 and calnexin in interactomes of IL-22BPi1 and IL-22BPi2. Silver-stained (S.S) gels of proteins (co-)immunoprecipitated (IP) with FLAG resins from HEK293 or HeLa cells transiently transfected with the indicated expression plasmids or control empty vector (EV) are shown. Numbered arrow-heads indicate proteins identified by mass spectrometry included in the inset summary Table (detailed information in Supplementary Data 2). Co-immunoprecipitated proteins without DSP treatment were immunoblotted for detection by FLAG Ab. Asterisk, IL-22BPi1; empty arrowhead, IL-22; solid rhombus, IL-22BPi2; empty rhombus, IL-2EX. (Inset) Summary table with the mass spectrometry results from protein identification. # Unique peptides, # Peptides, and # PSMs are: the number of peptide sequences unique to a protein group, the number of distinct peptide sequences in the protein group and the total number of identified peptide sequences (peptide spectrum matches) for the protein. **(C)** IL-22BPi1, IL-22BPi2 and IL-2EX4 bind the substrate-binding domain of GRP78. HEK293 cells were transiently transfected with the indicated constructs and 24 h later were either treated with 100 ng/ml Mut SubAB or SubAB for 3 h. Cell lysates (CL) were co-immunoprecipitated with FLAG agarose. CL and IP proteins were immunoblotted for FLAG or GRP78. **(D)** HEK293 cells were individually transfected with expression plasmids for the three IL-22BP isoforms together with either GRP78 or GRP78^T37G^ expression vectors or empty vector (EV). 24 h later secreted protein was measured by ELISA for IL-22BP (mean ± SEM; *n* = 3; ^*^p < 0.05, ^**^p < 0.01 by unpaired *t*-test).

In order to identify proteinaceous factors that may be responsible for the intracellular retention of IL-22BPi1, we analyzed the interactomes of the IL-22BP isoforms purified from Hela and HEK293 cells by nLC MS/MS. A 75-kDa band was co-purified from HEK293 cells, both with IL-22BPi1 and IL-22BPi2 but not IL-22BPi3, that was identified as GRP78. We observed that upon densitometric analysis of silver stained gels, the relative amount of GRP78 co-purified with IL-22BPi1 was more than 4 times higher than that associated with IL-22BPi2 (Figure 4A). In HeLa or HEK293 cells, we reproducibly identified by mass spectrometry 4 partners for IL-22BPi2, i.e., GRP78, calnexin, GRP94 and GRP170, all of which are ER-localized chaperone-type proteins (Figures 4A,B, *inset* and Supplementary Data 2). In immunoblot validations, interaction of IL-22BPi1 and IL-22BPi2 with GRP78, GRP94, GRP170, and calnexin was seen, as well as with additional ER residential proteins (Supplementary Figure 5). GRP78 was also identified as the major interacting partner of IL-2EX4 but was not co-purified with IL-2 or IL-22 (Figure 4B). GRP78 is an ER luminal HSP70-type chaperone that binds newly synthesized proteins during translocation into the ER, and contains two functional domains, a nucleotide-binding domain (NBD) that binds ATP, and a substrate-binding domain (SBD) that binds incompletely folded client proteins. In order to find out whether the presence of the extra sequence in IL-22BPi1 influenced the site of GRP78 with which it interacts, we treated cells with subtilase (SubAB), a cytotoxin which is known to favor cleavage of newly synthesized GRP78 to produce a 28-kDa C-terminal fragment containing the SBD and an unstable 44-kDa N-terminal fragment containing the NBD of GRP78 (37). Based on studies of immunoglobulin (Ig) synthesis, it is known that the conformation of the SubAB-cleaved GRP78 SBD changes in absence of its regulatory NBD, and this induces locked-in association of the SBD with newly synthesized Ig light chains (38). Figure 4C shows co-purification of full-length endogenous GRP78 and 28-kDa SubAB-cleaved GRP78 SBD bands with IL-22BPi1, IL-22BPi2, IL-2EX4 but not IL-22BPi3 demonstrating that any of these interactions occur with the SBD. Next, we co-transfected the three IL-22BP isoforms with wild-type (*wt*) GRP78 or a mutant form of GRP78, in which substitution of Thr-37 with Gly (T37G) inhibits ATPase activity and blocks ATP-mediated release of cargo from GRP78 (39). *Wt* but not GRP78^T37G^ significantly increased secreted levels of IL-22BPi1 and IL-22BPi2, while it did not affect secretion of IL-22BPi3 (Figure 4D). This was mirrored by a tendency toward increased intracellular levels of IL-22BP isoforms in the presence of *wt*GRP78 (Supplementary Figure 5). Thus, the binding of IL-22BPi1 and IL-22BPi2 to the SBD of GRP78 as well as their enhanced secretion upon overexpression with *wt* but not ATPase-deficient GRP78 suggests that both isoforms are natural client proteins of GRP78.

### IL-22BPi1 and IL-22BPi2 Interact With the C-Terminal Half (Amino Acids 455-577) of the Middle Domain of GRP94

**Figure 5:**
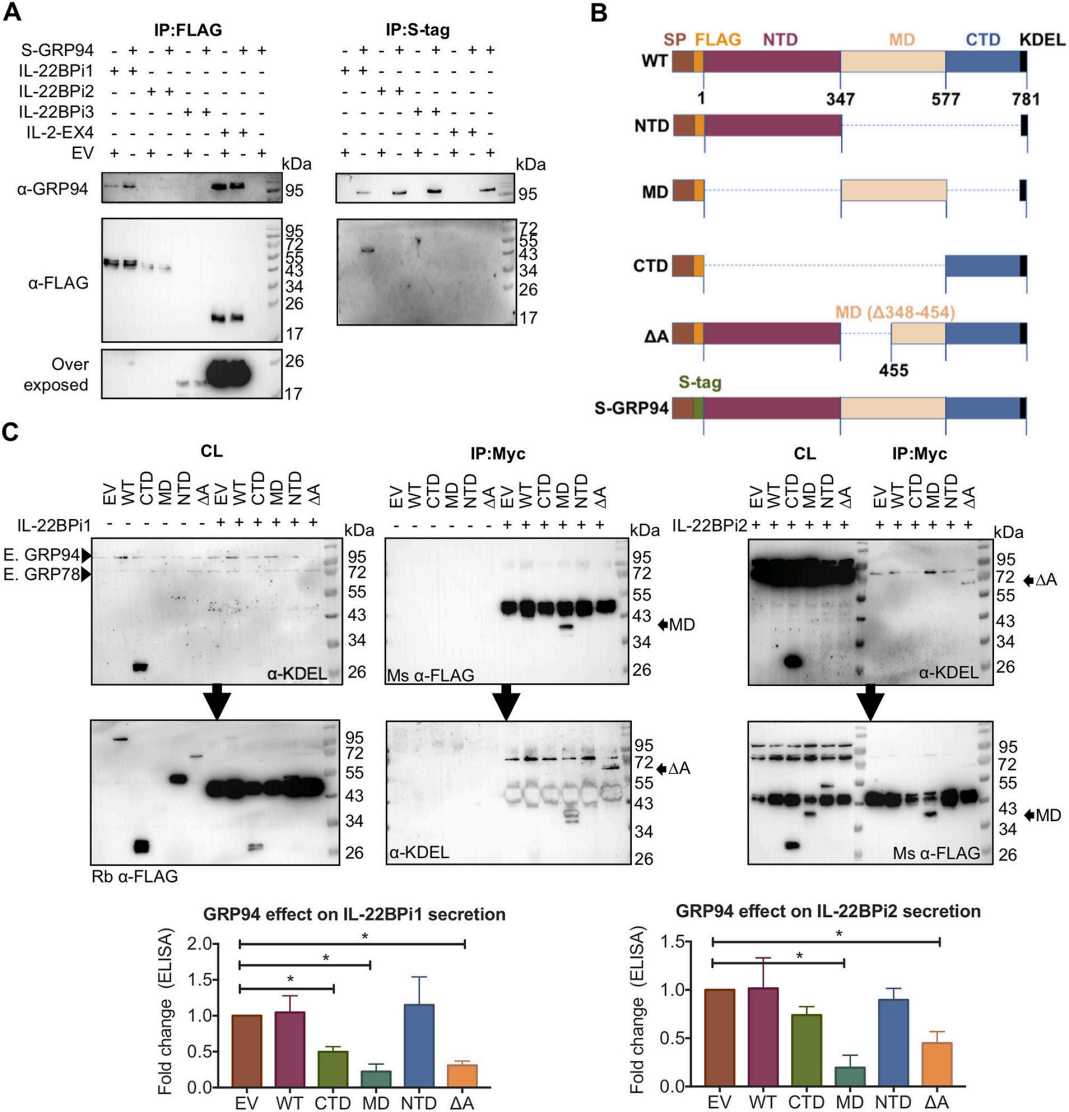
Both IL-22BPi1 and IL-22BPi2 interact with the middle domain of GRP94. **(A)** HEK293 cells were transiently co-transfected with the indicated expression plasmids, 24 h later cell lysates were immunoprecipitated with FLAG resin to IP IL-22BP isoforms or IL-2EX4, or with S-tag agarose to IP GRP94. (Co-)immunoprecipitated proteins (IP) were immunoblotted for GRP94 and FLAG. **(B)** A schematic diagram of full-length (WT) and structural mutants of GRP94 used. **(C)** (Western blot images) HEK293 cells were transiently co-transfected with the indicated expression plasmids, 24 h later cell lysates (CL) were co-immunoprecipitated with Myc agarose. Membranes with CL and IP proteins were sequentially exposed for detection by KDEL and mouse (Ms) or rabbit (Rb) FLAG Abs, or vice versa. MD-GRP94 or ΔA-GRP94 co-immunoprecipitated with IL-22BPi1 or IL-22BPi2 are indicated with black arrows. (Lower) Expression vectors for IL-22BP isoform-1 and−2 were individually transfected into HEK293 cells together with either GRP94 wild-type (WT) or GRP94 mutant vectors (CTD, MD, NTD, or deltaA) or empty vector (EV). Twenty-four hour after transfection, secreted IL-22BP was quantified by ELISA, analyzed by paired *t*-test on original data and represented as fold change relative to EV condition (mean ± SEM; *n* = 3; ^*^p < 0.05 by paired *t*-test, original paired data are represented in Supplementary Figure 6).

As shown above, both IL-22BPi1 and IL-22BPi2 interact with GRP94. Like GRP78, GRP94 is an ER luminal chaperone, but unlike GRP78, it has a highly selective client base of proteins, including specific Toll-like receptors and integrins, which apart from exhibiting disulfide-bonds share no further apparent structural features (40, 41). The amount of GRP94 co-IPed via anti-FLAG purification of IL-22BP isoforms from HEK293 cells transfected with identical quantities of cytokine vectors was relatively higher for IL-2EX4 than for IL-22BPi1; GRP94 was borderline detectable in IL-22BPi2 IP (Figure 5A, left panel). Upon reverse IP using S-tagged GRP94, IL-22BPi1 but not IL-22BPi2 nor BPi3 were found in complex with GRP94. We were unable to IP S-GRP94 from cells co-transfected with IL-2EX4 (Figure 5A, right panel). To identify the region(s) in GRP94 responsible for binding to IL-22BPi1 or IL-22BPi2, we probed interaction of both isoforms with a series of GRP94 truncation and deletion mutants. This selection of constructs was based on well-defined GRP94 regions (40) that constitute the modular fold of GRP94, and was previously used to investigate the interaction site of GRP94 with OS-9 (42) (Figure 5B). IL-22BPi1 or IL-22BPi2 were purified from cell lysates by means of their myc-tag. Although all GRP94 constructs contained both N-terminal FLAG-tag and C-terminal native KDEL sequences, their individual detectability in western blot varied according to the antibody used; mouse anti-FLAG Ab detected the GRP94 middle domain (MD) mutant better than did rabbit anti-FLAG Ab, and mouse anti-KDEL Ab was only successful in detecting the GRP94 C-terminal domain (CTD) and ΔA-GRP94 mutants (Figure 5C). CTD and N-terminal domain (NTD) GRP94 mutants were not found in complex with either isoform while the MD-GRP94 and ΔA-GRP94 MD variants interacted strongly with IL-22BPi1 and IL-22BPi2 (Figure 5C). Thus, while the middle subdomain of GRP94 shared by MD- and ΔA-GRP94 (amino acids 455-577) emerges as critical region for binding both IL-22BPi1 and IL-22BPi2, this client-binding site differs from that used by the ERAD component OS-9 [i.e., amino acids 356–456 of the GRP94 MD (42)] or by GRP94 client proteins such as TLRs and integrins [i.e., amino acids 635–656 of the GRP94 CTD; (41)]. Co-expression of either GRP94 MD or ΔA mutants with IL-22BPi1 or BPi2 isoforms significantly decreased their secretion, suggesting that interaction of these IL-22BP isoforms with these GRP94 mutants sequesters them from the productive folding/secretory pathway (Figure 5C, bottom graphs; Supplementary Figure 6). Truncated regions absent in MD and ΔA-GRP94 seem thus to be responsible for a proper GRP94 – IL-22BP interaction cycle. While the GRP94 NTD mutant did not affect secretion, the GRP94 CTD mutant appeared to decrease secretion of IL-22BPi1 but not that of IL-22BPi2, and this possibly reflects additional weak interactions of IL-22BPi1 with this domain that did not survive the IP procedure.

**Figure 6:**
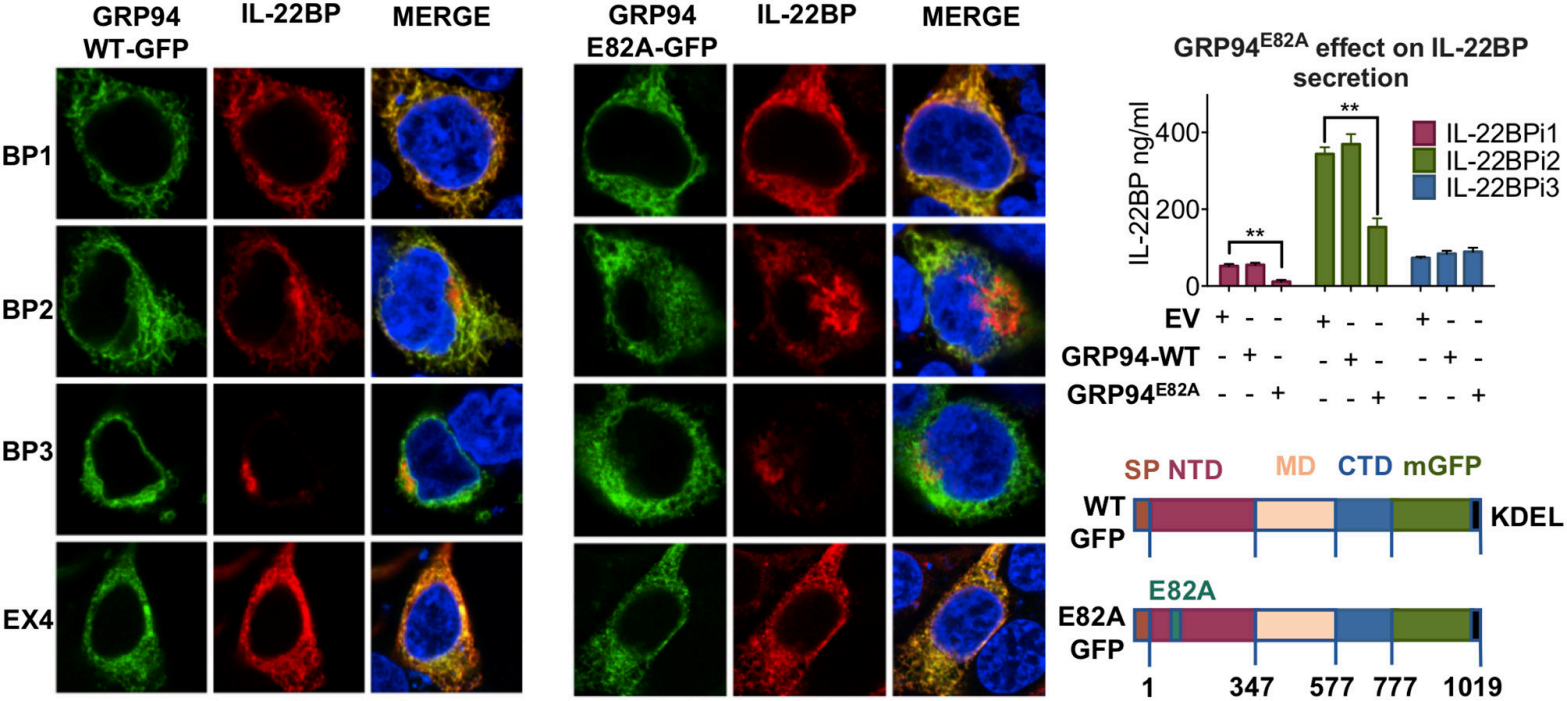
GRP94 ATPase activity is important for IL-22BPi1 and IL-22BPi2 secretion. GRP94 co-localizes with IL-22BPi1 and IL-22BPi2 but not IL-22BPi3. (Left) Confocal microscopy of HEK293 cells co-transfected with IL-22BP isoforms with the indicated GFP-GRP94 fusion constructs (depicted lower right); IL-22BP was stained using an anti-IL-22BP antibody (red, IL-22BP Ab 4), IL-2EX4 was stained with mouse anti-FLAG Ab, and the nucleus was stained using DAPI (blue). Secreted IL-22BP was measured by ELISA in the conditioned media (CM) of HEK293 cells transfected with individual expression vectors for IL-22BP isoforms together with the indicated GRP94 vectors (upper right) (mean ± SEM; *n* = 4; ^**^p < 0.01 by unpaired *t*-test).

Co-expression of GFP-tagged *wt*GRP94 as well as the ATPase-negative mutant GRP94^E82A^ (43) with IL-22BP isoforms revealed pronounced co-localization of either GRP94 form with IL-22BP1 and IL-2EX4 as well as with the reticular portion of IL-22BPi2 located outside the Golgi apparatus (see also Figure 2B), but not with IL-22BPi3 (Figure 6). Furthermore, compared to *wt*GRP94, GRP94^E82A^ significantly reduced secretion levels of IL-22BPi1 and IL-22BPi2, but not that of IL-22BPi3. Together, these experiments provide evidence that GRP94 interacts with both the former isoforms, and that the secretion of both isoforms is proportional to intact GRP94 activity.

### *IL22RA2* Alternatively Spliced Exon-Coded Sequence Confers Ability to Induce the UPR Program

**Figure 7:**
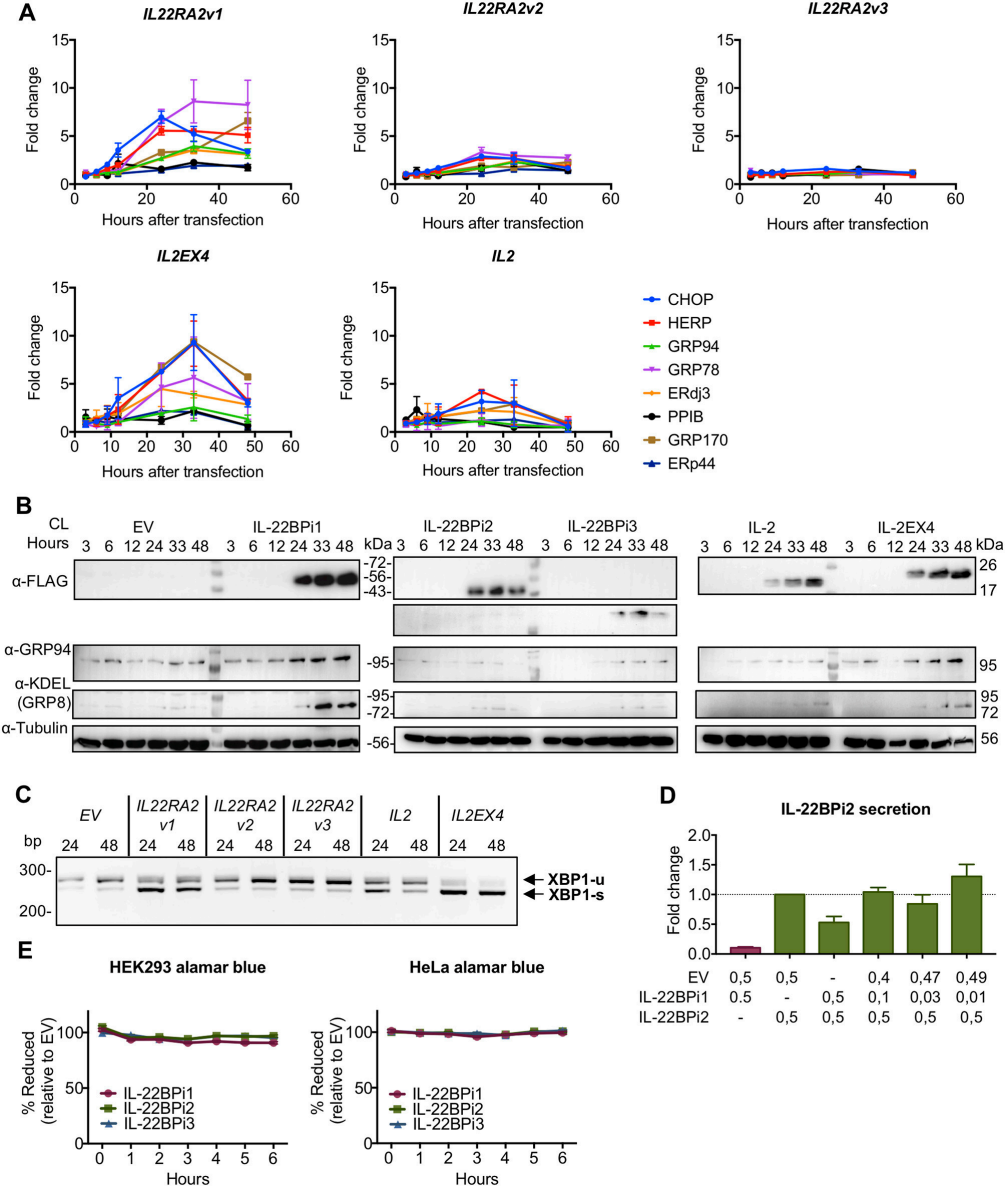
IL-22BPi1 and IL-2EX4 induce unfolded protein response (UPR) genes. HEK293 cells were transiently transfected with IL-22BPi1, IL-22BPi2, IL-22BPi3, IL-2, IL-2EX4 or empty vector (EV) as control. Cells were collected at the indicated hours after transfection. **(A)** Expression of different genes related to ER function or UPR were analyzed by RT-qPCR. Each gene expression value is represented as fold change relative to the same time-point expression value of the EV condition and relative to the housekeeping gene *GAPDH*. Mean ± SEM of three independent experiments. All primers are listed in Supplementary Table 2. **(B)** GRP78 and GRP94 protein levels correlate with mRNA levels observed in **(A)**. Cell lysates (CL) were immunoblotted for FLAG, GRP94, KDEL and tubulin as loading control. **(C)** IL-22BPi1 and IL-2EX4 cause XPB1 splicing. XBP1 splicing was detected with conventional PCR for the indicated conditions and times. Un-spliced and spliced XBP1 are indicated as XBP1-u or XBP-1s respectively. **(D)** IL-22BPi2 secretion was not increased when co-expressed with different ratios of IL-22BPi1. HEK293 cells were co-expressed with different ratios of EV:IL-22BPi1:IL-22BPi2 expression plasmids. 48 h later, secreted IL-22BP in conditioned media (CM) was quantified by ELISA (mean ± SEM; *n* = 3). **(E)** Cell viability measured with alamarBlue was not compromised by any of the conditions in two different cell lines. Reduction of alamarBlue was measured after 48 h of transfection and assayed for the indicated times and cell lines. Values are represented as percentage of reduction in each condition relative to EV (mean ± SEM; *n* = 3).

This data provides evidence for IL-22BPi1 being an IL-22 non-binding protein with stronger intrinsic propensity to misfold than IL-22BPi2. Rather than changing specificity of intracellular protein interactions based on interactome analysis, the alternatively spliced exon-encoded sequence appears to compromise folding and secretion of its recipient protein. Thus, both IL-22BPi2 and IL-22BPi1 are client proteins of, and interact with identical domains of, GRP78 and GRP94, but IL-22BPi1 binds more strongly to each of these than IL-22BPi2, is less efficiently secreted, accumulates intracellularly, and is apparently confined to the luminal ER seen via immunofluorescence co-localization with GRP94 and ERp72 coupled to less protein accumulation in the Golgi apparatus than that seen for IL-22BPi2 and BPi3. We tested whether expression of IL-22BPi1 activates the unfolded protein response (UPR), a resolutive transcriptional program to maintain ER homeostasis (44), that is activated in response to accumulation of misfolded proteins in the ER. Figure 7A shows that transfection of HEK293 cells with IL-22BPi1 or IL-2EX4 induces expression of typical UPR genes including GRP78, GRP94, CHOP, and HERP peaking at around 36 h after transfection. In contrast, IL-22BPi2, BPi3, or IL-2 had much less or no effect on induction of these UPR genes.

Moreover, analysis of cell lysates in western blot revealed that appearance of IL-22BPi1 or IL-2EX4 at 24 h after transfection is associated with increased protein levels of GRP94 and GRP78 (Figure 7B). No such effects were seen with IL-22BPi2, IL-22BPi3, or IL-2. Activation of the UPR was confirmed by enhanced splicing of XBP1 in cells expressing IL-22BPi1 or IL-2EX4 (Figure 7C). Given the observation that ectopic GRP78 enhanced secretion of both IL-22BPi2 and BPi1 (see Figure 4D), we analyzed whether co-transfection of IL-22BPi2 with IL-22BPi1 may, similarly, enhance IL-22BP secretion secondary to UPR induction of GRP78 by IL-22BPi1. Figure 7D shows that at a BPi1:BPi2 1:1 transfection ratio, reduced secretion of IL-22BP measured in ELISA was seen, while at smaller ratios approaching those seen in natural producer cells (see Figure 1) at best a non-significant trend toward enhanced secretion of immunoreactive protein was observed (same effect was observed when IL-22BPi2 was co-transfected with IL-2 or IL-2EX4 rather than IL-22BPi1, Supplementary Figure 7). We assayed if UPR activation compromised cell viability in this experiment, but this was not the case (Figure 7E).

### Expression of IL-22BP Affects GRP78 Levels in Immature moDCs

**Figure 8:**
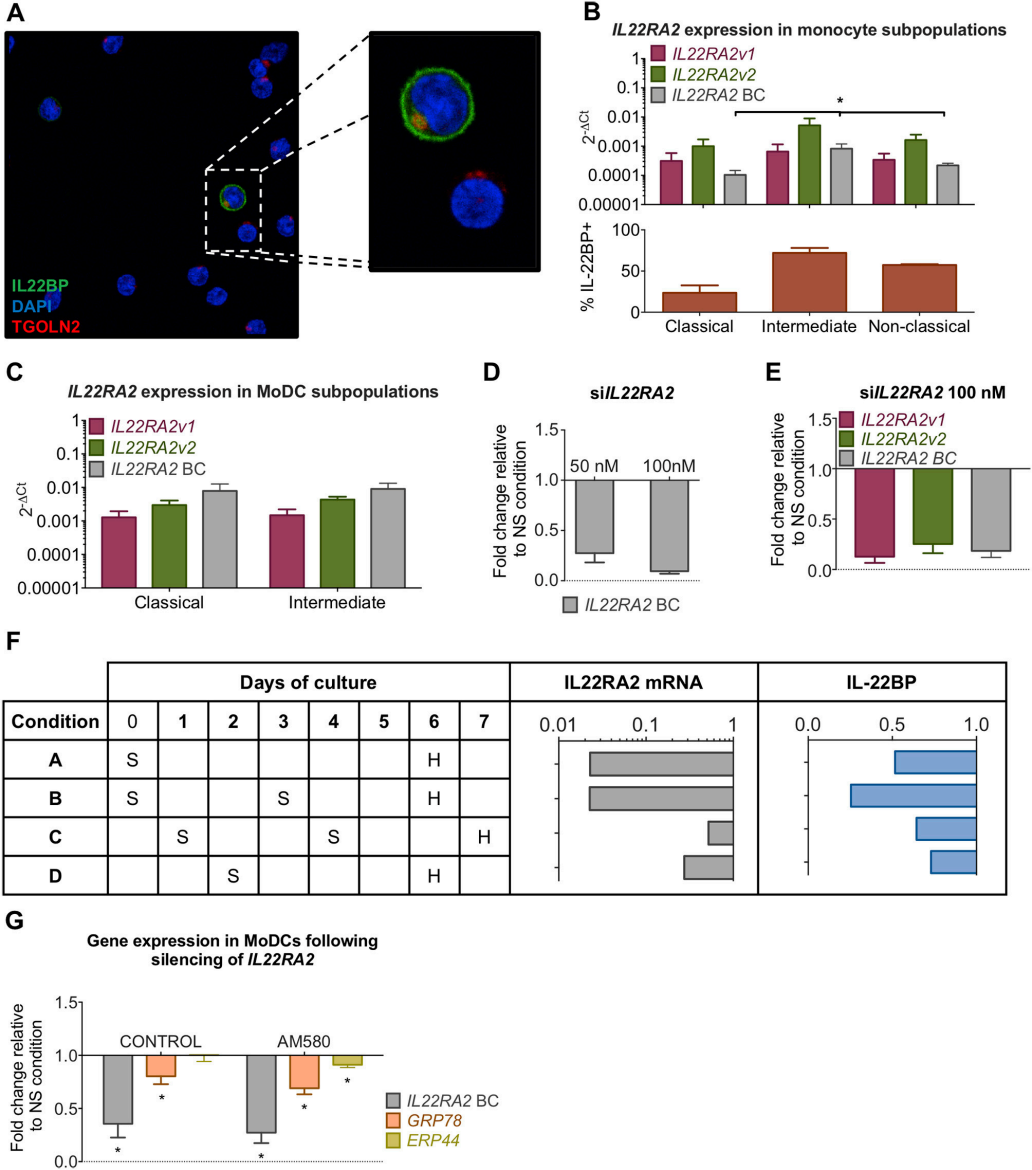
Silencing of *IL22RA2* in immature moDCs reduces GRP78 expression. **(A)** CD14^+^ monocytes were stained with DAPI (blue), trans-GOLGI (red) and IL-22BP (green, IL-22BP Ab 4). **(B)** Overall (best coverage, BC) and isoform-specific *IL22RA2* expression levels in classical, intermediate and non-classical monocyte subpopulations (top) from four healthy donors (average ± SEM). Percentages of IL-22BP^+^ monocytes found in each subpopulation by immunofluorescence microscopy (bottom) (mean ± SEM; *n* = 4; ^*^p < 0.05, Kruskal-Wallis test for comparison of differences in *IL22RA2* mRNA levels or IL-22BP^+^ counts in the three subpopulations). **(C)** *IL22RA2* expression levels in classical and intermediate immature moDCs at day 6 of cultivation. moDCs derived from non-classical monocytes showed high levels of cell death and had not experienced any changes in the shape of the cells. High Ct values of HK gene *HPRT1* in qPCR compromised correct interpretation of *IL22RA2* levels in this subgroup, which was therefore excluded from the graph. **(D)** Fold change relative to non-targeting siRNA control of *IL22RA2* expression in CD14^+^ monocyte.-derived immature DCs on day 5 of cultivation following *IL22RA2* silencing with Viromer Green at two concentrations of siRNA. **(E)** Silencing as described in **(D)** was not variant specific and both *IL22RA2v1* and *v2* were silenced to similar extent. **(F)** Scheme comparing efficiencies of four distinct silencing strategies on suppression of *IL22RA2* mRNA (qPCR) and intracellular IL-22BP (ELISA) in immature CD14^+^ moDCs using 100 nM *IL22RA2* siRNA or non-targeting siRNA in combination with Viromer Green. S, silencing, H, harvest. Bar diagrams represent fold change relative to non-targeting siRNA. **(G)** Effect of *IL22RA2* silencing on GRP78 and ERp44 mRNA levels in CD14^+^ monocyte-derived immature DCs from healthy donors using silencing strategy B in **(F)**. CD14^+^ monocytes were cultivated in DM medium in the absence or presence of AM580 starting on day 0 up to the day of harvest. Average of fold change (±SEM) relative to non-silencing control over 6 (control) and 5 (AM580) independent measurements relative to the housekeeping gene HPRT1. ^*^p < 0.03 by Wilcoxon test.

We asked whether the link between IL-22BPi1 and GRP78 induction is active in natural producer cells. If so, reduction of IL-22BP expression levels by silencing should decrease GRP78 levels in monocyte-derived DC cells. Prior to this experiment, we had noted that a proportion of monocytes purified from PBMCs without prior cultivation, around 10%, stained intensely positively for IL-22BP upon immunofluorescence microscopy, suggesting ongoing IL-22BP synthesis in a subset of unprimed and unstimulated monocytes of healthy donors (Figure 8A). Since three types of monocytes in the blood are commonly distinguished on the basis of CD14 and CD16 surface expression (45) (i.e., classical, CD14^++^ CD16^−^; non-classical, CD14^+^CD16^++^; intermediate, CD14^++^CD16^+^), we analyzed whether differential expression of IL-22BP in these types may explain the observed microscopic discrepancies. Analysis of *IL22RA2* and cytokine expression in purified CD16^−^CD14^+^ and CD16^+^ monocyte subpopulations, and their corresponding immature or IFN-γ/LPS-matured dendritic cells showed higher expression of *IL22RA2* in CD16^+^ monocytes and CD16^+^ monocyte-derived immature DCs compared to the respective CD16^−^CD14^+^ subsets (Supplementary Data 1). Classical, intermediate and non-classical monocytes were subsequently separated by FACS, and *IL22RA2* expression was analyzed in each subtype (Gating strategy in Supplementary Figure 1). Though all three monocyte types expressed similar ratios of *IL22RA2v1* vs. *IL22RA2v2* mRNA, Figure 8B shows that intermediate CD14^++^CD16^+^ monocytes produced the highest overall levels of *IL22RA2* and isoform-specific *IL22RA2v1* and *v2* mRNAs. This was validated on the protein level by direct counting of IL-22BP^+^ cells in immunofluorescence microscopy (Figure 8B, lower panel). Next, we differentiated the three monocyte types into immature moDCs. At day 6, the non-classical type showed extensive cell death in line with a previous report and was excluded from further analysis (46). However, in contrast to the primary uncultivated monocytes, classical and intermediate moDCs showed higher and reciprocally similar levels of *IL22RA2v1* and *v2* mRNA (Figure 8C). In silencing experiments, therefore, moDCs derived from CD14^+^ unfractionated monocytes were used. Using a polymer-based transfection reagent (i.e., viromer), we achieved over 85% reduction of *IL22RA2* mRNA levels using 100 nM siRNA, with both *IL22RA2v1* and *v2* reduced to a similar extent (Figures 8D,E). Following comparison of various silencing procedures, we found that silencing on day 0 of the cultivation period, repeated on day 3, followed by harvest on day 6, resulted in the most pronounced reduction of both *IL22RA2* mRNA and intracellular IL-22BP protein measured by ELISA (condition B in Figure 8F). Silencing done on day 1 or 2 was much less effective condition C and D in Figure 8F), while a unique silencing on day 0 was less effective in reducing IL-22BP protein (condition A in Figure 8F). In none of these conditions did *IL22RA2* silencing affect cell viability measured by alamarBlue. Condition B was applied to moDCs from unrelated healthy donors, co-cultivated or not with AM580. Measured by qPCR, *IL22RA2* and *GRP78* mRNA levels were significantly reduced in both conditions, while *ERP44*, a gene less strongly affected by IL-22BPi1-induced UPR in HEK293 cells [(47); see also Figure 7A), was reduced to a lesser extent and only significantly so in AM580-treated moDCs (Figure 8G). Thus, *IL22RA2* expression level in immature moDCs influences *GRP78* expression level.

## Discussion

IL-22BPi1 differs from IL-22BPi2, a secreted high-affinity IL-22 antagonist, by the insertion of a 32-amino acid sequence coded for by an alternatively spliced exon. This insertion occurs at position 67 of IL-22BPi2 (231 amino acids) and does not disrupt the reading frame as a consequence of which the intact primary sequence of IL-22BPi2 is in its entirety included in IL-22BPi. The biological function of IL22BPi1 is not known. Here, we show that IL-22BPi1 displays hallmarks of a poorly secreted, misfolded protein incapable of binding IL-22 in biological or co-folding assays. IL-22BPi1, but not IL-22BPi2 or BPi3, induced a UPR response resulting in higher GRP78 and GRP94 protein levels. Silencing of IL-22BP in immature CD14^+^ monocyte-derived DCs reduced GRP78 expression. This study describes a novel intracellular function of IL-22BPi1 unrelated to neutralization of IL-22.

In immature moDCs, the human *IL22RA2* gene co-expresses mRNAs for three protein isoforms that share the same signal peptide and should therefore be secreted. In line with earlier observations, treatment of moDCs with retinoic acid receptor agonist AM580 increases, while maturation with IFN-γ and LPS strongly suppresses *IL22RA2* mRNA levels (5). Though discernible by ELISA, IL-22BP secreted by moDCs was poorly detected in CM or acetone precipitates via western blot. Thus, to determine the biochemical characteristics and secretory potential of each isoform we used HEK293 cells transfected with individual expression vectors for IL-22BPi1, IL-22BPi2 or IL-22BPi3. Similar to Lim and colleagues (12), we were unable to detect IL-22BPi1 in concentrated culture medium of transfected cells by western blot, though we could discern small amounts of secreted protein by ELISA. Subcloning of the alternatively spliced exon from IL-22BPi1 into IL-22BPi3 arrested the latter's secretion (12). In this work, insertion of this exon into the reading frame of IL-2 (IL-2EX4), an abundantly secreted cytokine, was sufficient to block its secretion. IL-22BPi2 and IL-22BPi3, but not IL-22BPi1, were mainly visible as intensely staining deposits co-localizing with a trans-Golgi protein marker. The diffuse perinuclear staining pattern of IL-22BPi1, in contrast, resembled that of the luminal ER protein ERP72, suggesting that the extra sequence acts as a powerful ER retention signal limiting transit toward the Golgi apparatus. We set out to determine the factors that cause intracellular retention of IL-22BPi1. GRP78, a crucial luminal ER chaperone involved in translocation, folding and retrograde transport of newly synthesized proteins via direct interaction with the polypeptide backbone (48) was identified in the interactome analysis of the affinity-tagged IL-22BP isoforms purified from total cell lysates as main interacting partner of IL-22BPi1, IL-22BP2, and IL-2EX4. These three proteins, but not IL-22BPi3, were found to interact with the SBD of GRP78. Co-transfection with *wt*GRP78, but not the ATPase-negative T37G GRP78 mutant (39) significantly increased secretion of IL-22BPi1 and BPi2, but not that of BPi3, as measured in ELISA. Thus, both IL-22BPi1 and IL-22BPi2 appear to behave as *bona fide* secretory client proteins of GRP78, and the extra exon sequence does not appear to change site of interaction with GRP78. Interactomes of IL-22BPi2 purified from either HEK293 or HeLa cells reproducibly contained, apart from GRP78, also calnexin, GRP170 and GRP94, suggesting extensive ER quality control scrutiny of its folding. These chaperones, as well as ERdj3, were found to interact with both IL-22BPi2 and IL-22BPi1 in western blot analysis. The C-terminal half (amino acids 455-577) of the middle domain of GRP94 was identified as critical client binding region of both IL-22BPi1 and BPi2. This region differs from that identified for the GRP94 clients OS-9 or TLRs and integrins (41, 42), but the implications are as yet difficult to interpret given the scarcity of information on the mechanisms defining interaction of client proteins with GRP94. The ATPase-negative GRP94 MD and ΔA mutants that show strong interaction with both isoforms, also reduced their secretion, indicative for competition with a productive “pro-secretion” role of GRP94 in IL-22BPi1 or IL-22BPi2 folding dependent on functionally intact GRP94. IL-22BPi1 and IL-2EX4 showed virtually perfect co-localization with GRP94 identifying this chaperone as crucially associated with their arrest of transit to Golgi. Calnexin is part of the ER quality control system that acts to retain unfolded *N*-linked glycoproteins (49), compatible with mono-glucosylation of the high-mannose type *N*-glycans we identified on intracellular IL-22BP isoforms. GRP170, an HSP70-type chaperone, and ERdj3 a DnaJ-family GRP78 co-factor, recognize distinct sets of peptide sequences in unfolded proteins; interestingly, while GRP78 and ERdj3 can interact with many diverse client peptide sequences, GRP170 was shown recently to interact specifically with rarer, aggregation-prone regions which, if continuously exposed, may trigger disposal of the protein by ERAD given the toxicity of protein aggregates to cell survival (50). This potential link to ERAD is substantiated by the observation that both IL22-BPi1 and IL-22BPi2, but not IL-22BPi3, appear to be partially degraded by the proteasome.

We investigated whether co-expression of IL-22BP isoforms with their ligand IL-22 may alleviate the formers degradation through enhanced secretion via assembly-induced folding. Such process has been documented in detail for IL-15 that is pre-assembled in complex with IL-15Rα in the ER/Golgi of DCs. IL-15Rα acts as a chaperone for unstable IL-15 inhibiting its degradation through the proteasome (32, 36). A different but related process takes place in eosinophils, where an intracellular pool of IL-4Rα enhances secretory trafficking of IL-4 for rapid mobilization into secretory vesicles prior to secretion (51). For IL-12 and IL-23, co-expression of the shared β-subunit (p40) inhibits misfolding of the poorly secreted α-subunits (p35 and p19, respectively) facilitating secretion of the functional heterodimers (52, 53). In line with these studies, we found that co-expression of IL-22 enhanced secretory transit of IL-22BPi2 and IL-22BPi3 resulting in much higher levels of secreted proteins. This was not seen for IL-22BPi1 of which secretion levels were unaffected by IL-22 co-expression. We were not able to detect expression of IL-22 in immature or mature moDCs by qPCR; thus, whether IL-22 – IL-22BPi2 assembly-induced folding takes place in any other natural producer cell types such as CD4^+^ T lymphocytes (10) remains to be determined. However, together with bioactivity measurements, which showed that IL-22BPi2 but not IL-22BPi1 when used at similar concentrations, inhibited STAT3 phosphorylation by IL-22, it appears that IL-22BPi1 is naturally incapable of interacting with IL-22. This is in accordance with the location of residues critical for binding of IL-22BPi2 to IL-22, especially Lys^65^ and Tyr^67^, close to the position of insertion of the alternatively spliced exon in IL-22BPi1 (15, 54). Dumoutier et al. (55) demonstrated that of the IL-10-type homologs including IL-19, IL-20, and IL-24, only IL-22 was able to bind to IL-22BPi2. These observations render the potential for any residual interaction between IL-10-type cytokines and IL-22BPi1 that may “rescue” its misfolding highly unlikely. We tested the capacity of IL-22BP isoforms to activate ER stress signaling via induction of UPR through accumulation of misfolded protein (56). IL-22BPi1 strongly upregulated expression of UPR genes resulting in increased GRP78 and GRP94 protein levels, suggesting that under these experimental conditions ERAD alone is insufficient to restore ER folding capacity (57). In contrast, IL-22BPi2 did not augment GRP78 and GRP94 protein levels indicating that its more balanced export from the ER through either secretion or ERAD may eliminate the need for UPR induction. Mirroring the findings in recombinant cells, silencing of *IL22RA2* expression in immature CD14^+^ monocyte-derived DCs coincided with reduced GRP78 expression.

The failure of IL-22BPi1 to fold may be partially due to the general tendency of alternatively spliced exons to encode intrinsically disordered protein segments (58). In fact, exonization processes may act as mechanisms to add disordered regions to proteins, and many of these play a role in protein-protein interactions (59). The alternatively spliced exon of IL22RA2v1 evolved from a retrotransposon of the mammalian apparent Long Terminal Repeat family (MaLR) specific to the great ape lineage that became active consequential to a single mutation in the proto-splice site (60). Computational structure prediction of the three mature IL-22BP isoforms shows that the only region with high intrinsic disorder profile is that coded for by this exon (Supplementary Figure 8). Moreover, the sequence and conformation of IL-22BPi2 have phenotypically evolved for high-affinity binding to and neutralization of IL-22. The presence of the extra exon in IL-22BPi1 breaks this selection pressure. We cannot exclude the possibility that specific beneficial interactions with IL-22BPi1 occur with as yet undetermined partners within natural producer cells or in the extracellular milieu. Our work suggests that one benefit of co-expression of IL-22BPi1 with IL-22BPi2 may reside in its ability through UPR induction to augment GRP78 and GRP94 levels. Since both these chaperones are instrumental in the folding of IL-22BPi2, enhanced secretion levels of IL-22BPi2 by moDCs could thus constitute a positive selectable trait. This will need to be investigated by abolishment of isoform-1 expression in moDCs through genome modification techniques such as CRISPR. Finally, pending further investigation, constitutively higher levels of ER chaperones may prepare the ER of immature moDCs for enhanced processing potential of secretory and membrane proteins including MHC receptors, integrins and cytokines which are massively expressed immediately upon receipt of TLR ligands or other maturation triggers (61).

## Author Contributions

PG-F, AU, IA, and KV designed and performed the experiments. PG-F, AU and KV wrote the manuscript. AP, JP, FB, DD, YA, and IA provided crucial reagents and/or helpful insights and critically reviewed the manuscript.

### Conflict of Interest Statement

The authors declare that the research was conducted in the absence of any commercial or financial relationships that could be construed as a potential conflict of interest.

## Supplementary Material

Presentation 1 **Supplementary Figure 1** (Strategy for monocyte subpopulation sorting); **Supplementary Figure 2** (Flow cytometric analysis of moDCs); **Supplementary Figure 3** (IL-22BPi2 detection with different antibodies); **Supplementary Figure 4** (Cell fractionation); **Supplementary Figure 5** (Protein partners of IL-22BPi1 and IL-22BPi2, WB-validation); **Supplementary Figure 6** (original paired values of secreted IL-22BP from Figure 5); **Supplementary Figure 7** (IL-22BPi2 secretion is not increased in presence of IL-2 or IL-2EX4); **Supplementary Figure 8** (Intrinsic protein disordered region prediction of IL-22BPi1); **Supplementary Table 1** (List of IL-22BP antibodies used throughout this study) and **Supplementary Table 2** (Primers used for gene expression and cloning).

Supplementary Data 1 Analysis of IL22RA2 and cytokine gene expression by qPCR and surface expression markers by flow cytometry in CD16^−^/CD14^+^ or CD16^+^ monocytes and their corresponding derived immature and mature dendritic cells.

Supplementary Data 2 Protein identified by mass spectrometry.
