# Supplementary material for: Long Interleukin-22 Binding Protein Isoform-1 Is an Intracellular Activator of the Unfolded Protein Response
Source: Front Immunol. 2018 Dec 14;9:2934. doi: 10.3389/fimmu.2018.02934 (PMC6302113; doi:10.3389/fimmu.2018.02934)
Supplement: Presentation 1 — Supplementary Figure 1 (Strategy for monocyte subpopulation sorting); Supplementary Figure 2 (Flow cytometric analysis of moDCs); Supplementary Figure 3 (IL-22BPi2 detection with different antibodies); Supplementary Figure 4 (Cell fractionation); Supplementary Figure 5 (Protein partners of IL-22BPi1 and IL-22BPi2, WB-validation); Supplementary Figure 6 (original paired values of secreted IL-22BP from Figure 5); Supplementary Figure 7 (IL-22BPi2 secretion is not increased in presence of IL-2 or IL-2EX4); Supplementary Figure 8 (Intrinsic protein disordered region prediction of IL-22BPi1); Supplementary Table 1 (List of IL-22BP antibodies used throughout this study) and Supplementary Table 2 (Primers used for gene expression and cloning). [file Presentation_1.pdf]

Supplementary Material

Long interleukin-22 binding protein isoform-1 is an intracellular activator of the unfolded protein response

Paloma Gómez-Fernández[1,2], Andoni Urtasun[1,2], Adrienne W. Paton[3], James C. Paton[3], Francisco Borrego[4,5,6], Devin Dersh[7,8], Yair Argon[7], Iraide Alloza[1,2] and Koen Vandenbroeck[1,2,6*]

*** Correspondence:** Koen Vandenbroeck k.vandenbroeck@ikerbasque.org

1 Supplementary Data

Supplementary Data 1. CD14^{+}/CD16^{+} monocyte purification and cytokine measurements

Supplementary Data 2. Information on protein identification

2 Supplementary Figures and Tables

2.1 Supplementary Figures

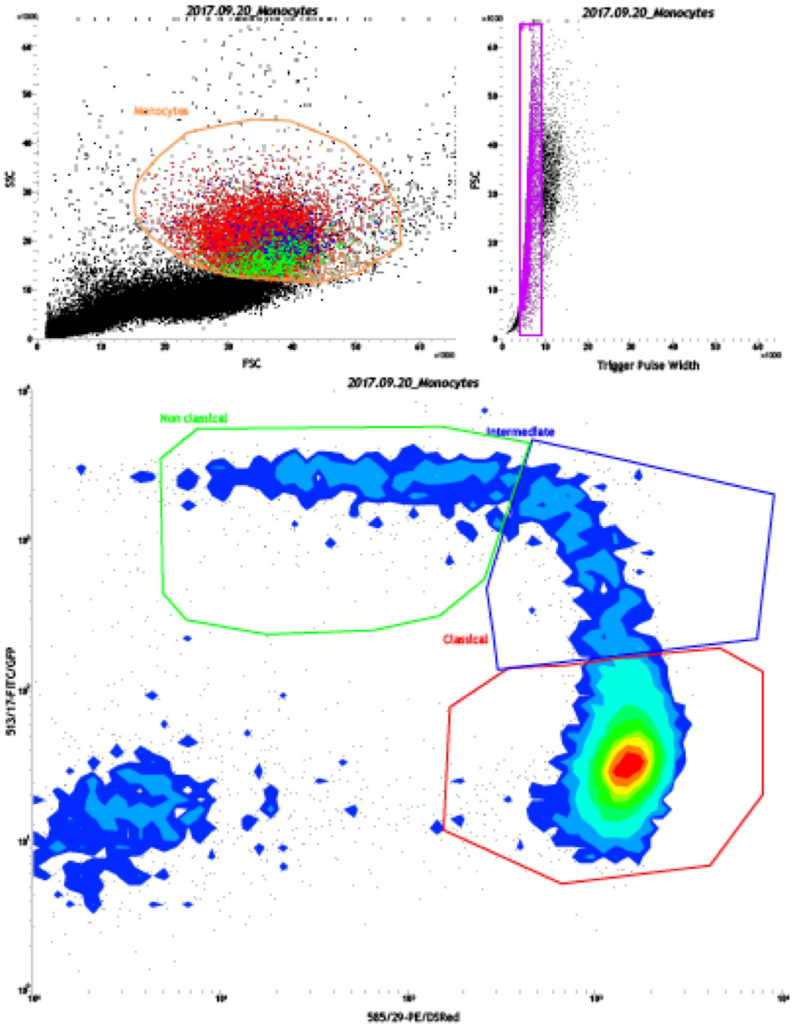

Supplementary Figure 1. Sorting strategy for monocyte subpopulation sorting. Sorting strategy consisted in a size-complexity discrimination of monocytes (orange circle, up left), selection of events with one cell width (purple, up right) and finally monocyte subpopulations discrimination based on CD14 (PE) and CD16 (FITC) labelling (bottom, density plot)

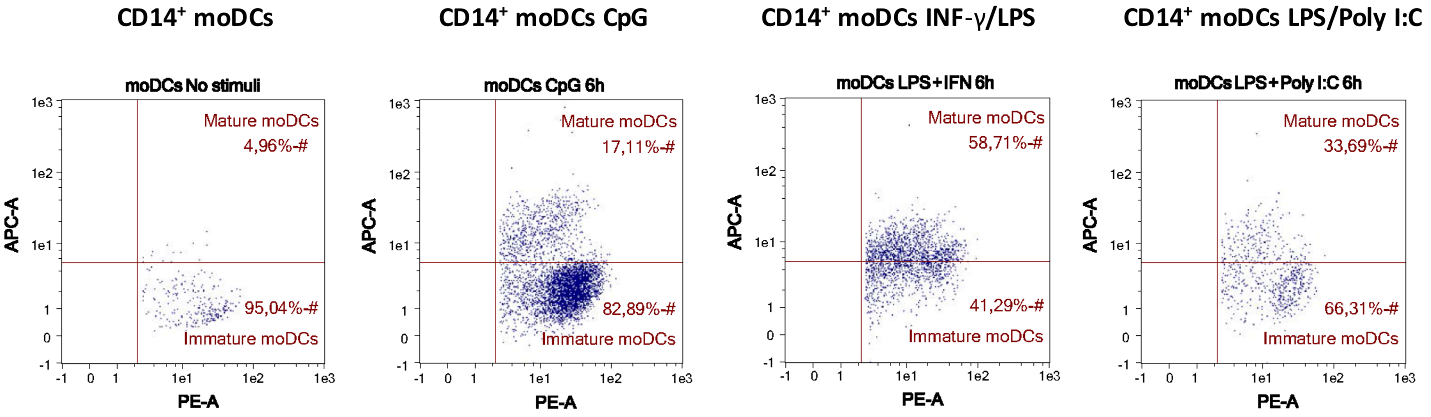

Supplementary Figure 2. Flow cytometric analysis of moDCs using Mo-DC Differentiation Inspector following use of different maturation stimuli. *CD83=APC*, *CD209=PE*.

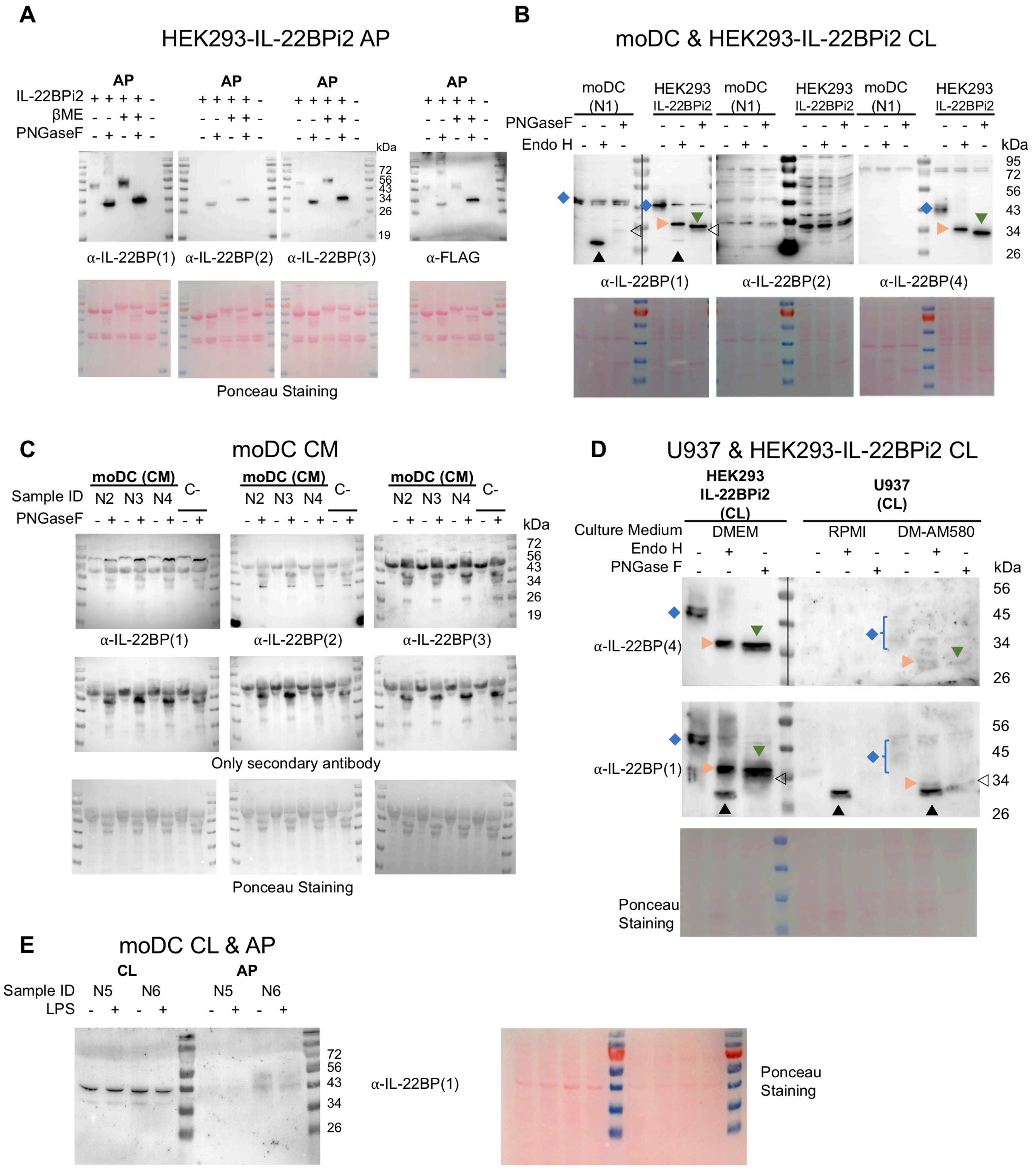

Supplementary Figure 3. IL-22BPi2 detection with different antibodies. **(A)** HEK293 cells were transiently transfected with the expression vector encoding IL-22BPi2 or left untransfected. 300 µl

of conditioned medium (CM) were acetone precipitated (AP), treated with or without PNGase F and immunoblotted for IL-22BP with different antibodies (S1 Table) under non- or reducing conditions (βME). Ponceau staining indicates equal loading volumes. PNGase F treatment enhanced detection of the secreted form of IL-22BPi2 by all four antibodies. **(B)** moDCs were harvested after 6 days in DM supplemented with AM580. HEK293 cells were transfected as in (A). Cell lysates (CL) were treated with or without PNGase F or Endo H and immunoblotted for detection of IL-22BP with different antibodies. Ponceau staining indicates equal loading quantity. Glycosylated IL-22BPi2, Endo H and PNGase F products are indicated, respectively, with a blue diamond, orange arrowhead or green inverted triangle; black triangles and open arrowheads indicate the reactivity of the antibody α-IL-22BP (1) for Endo H and PNGase F enzymes, respectively. Vertical line separates parts of the same membrane under different exposures (left part, long; right part, short). A 43-kD band reactive with α-IL-22BP antibody (1) in moDCs was partially deglycosylated. **(C)** moDC CM from three different healthy controls (N3, N4 and N5) was collected after 6 days in DM, 20 µl of each or culture media without cell contact (C-) were subjected to PNGaseF treatment to enhance detectability of secreted IL-22BP proteins (see A) and immunoblotted for detection of IL-22BP with three different α-IL-22BP antibodies. Before primary antibody incubation, membranes were immunoblotted with the corresponding secondary-HRP conjugated antibody. Notice the high antibody reactivity with the serum that is present in the CM. Ponceau staining indicate equal loading volumes. We did not detect specific proteins in the CM of moDC reactive with any of the three antibodies used. **(D)** U937 were harvested after 6 days of culture in RPMI or DM supplemented with AM580. HEK293 cells were transfected as in (A). Cell lysates were treated with or without PNGase F or Endo H and immunoblotted for detection of IL-22BP with different antibodies. Ponceau staining indicates equal loading quantity. Glycosylated IL-22BPi2, Endo H and PNGase F products are indicated, respectively, with a blue diamond, orange arrowhead or green inverted triangle; black triangle and open arrowheads indicate the reactivity of the antibody α-IL-22BP (1) for Endo H and PNGase F enzymes respectively. Anti-IL-22BP immunoreactive bands have higher Mr in HEK293 compared to U927 cells due to added myc-FLAG affinity tag. Vertical line separates parts of the same membrane under different exposures (left part, short; right part, long). **(E)** moDCs and CM were harvested after 6 days in DM during the last 20 hours of which cells were treated with LPS or left untreated. 300 µl of CM was AP-ed and immunoblotted for detection of IL-22BP under reducing or non-reducing conditions. Ponceau staining showed equal quantities and volumes loaded.

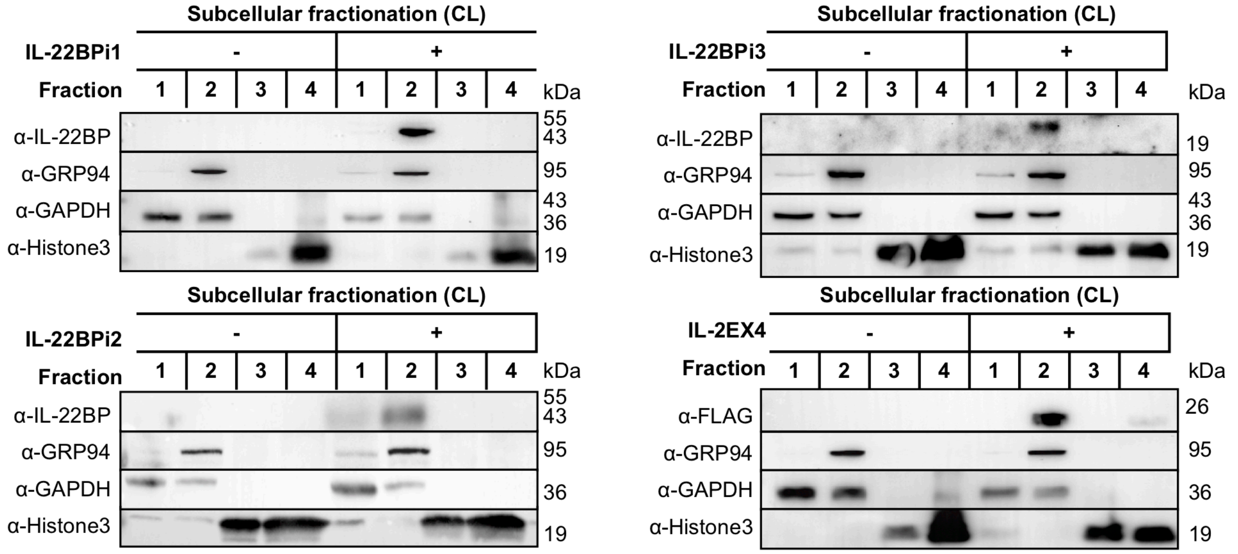

Supplementary Figure 4. IL-22BPi1, BPi2, BPi3 and IL-2EX4 are located in the membranous organelles fraction. HEK293 cells were transiently transfected with the indicated expression plasmids, and after 24 hours, cells were collected and fractionated following Holden and Horton's protocol (*1*). Equal amounts of protein per fraction were immunoblotted for GRP94, GAPDH, IL-22BP (Ab 4), FLAG and Histone3. Fractions 1, 2, 3 and 4 represent cytosol, membranous organelles, nucleus and insoluble fractions respectively.

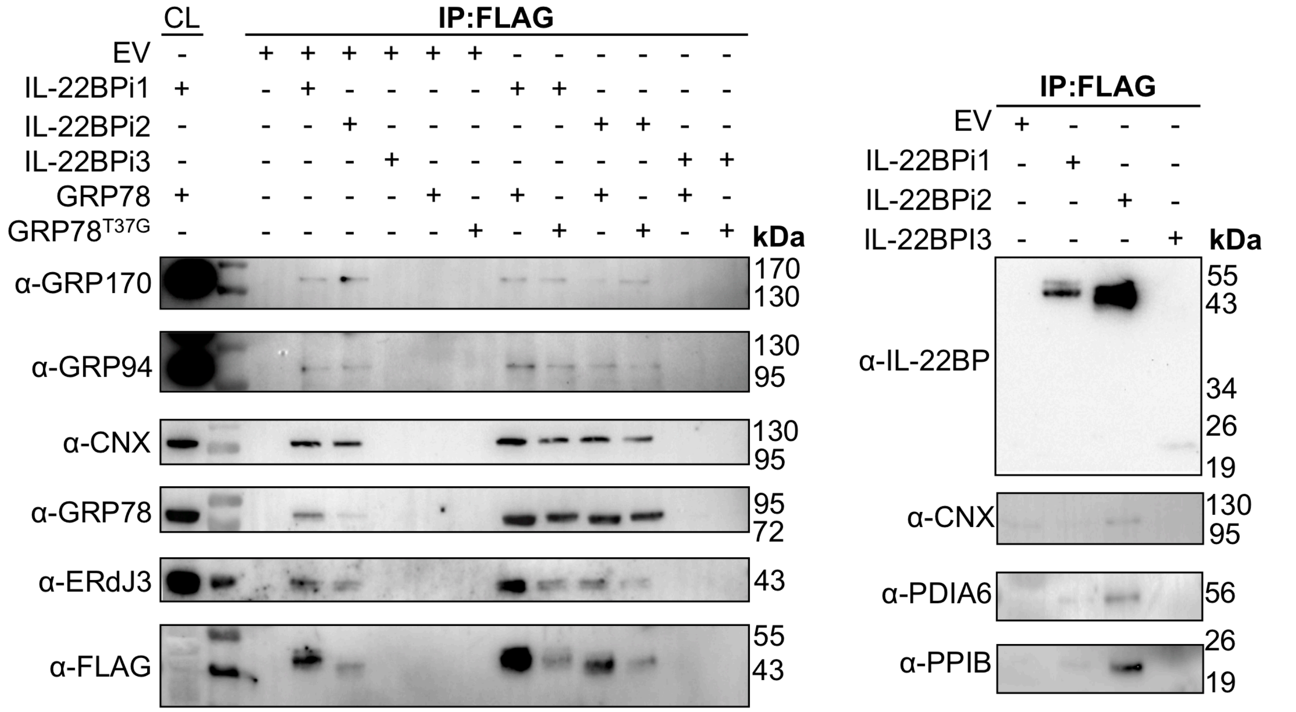

Supplementary Figure 5. GRP94, GRP78, PPIB, PDIA6, ERdj3, calnexin and GRP170 are partners of IL-22BPi1 and IL-22BPi2. HEK293 cells were transiently co-transfected with the indicated expression plasmids, after 24 hours cell lysates (CL) were co-immunoprecipitated with FLAG agaroses. Co-immunoprecipitated proteins (IP) were immunoblotted for GRP170, GRP94, calnexin (CNX), GRP78, ERdj3, PPIB, PDIA6, IL-22BP (Ab 4) and FLAG.

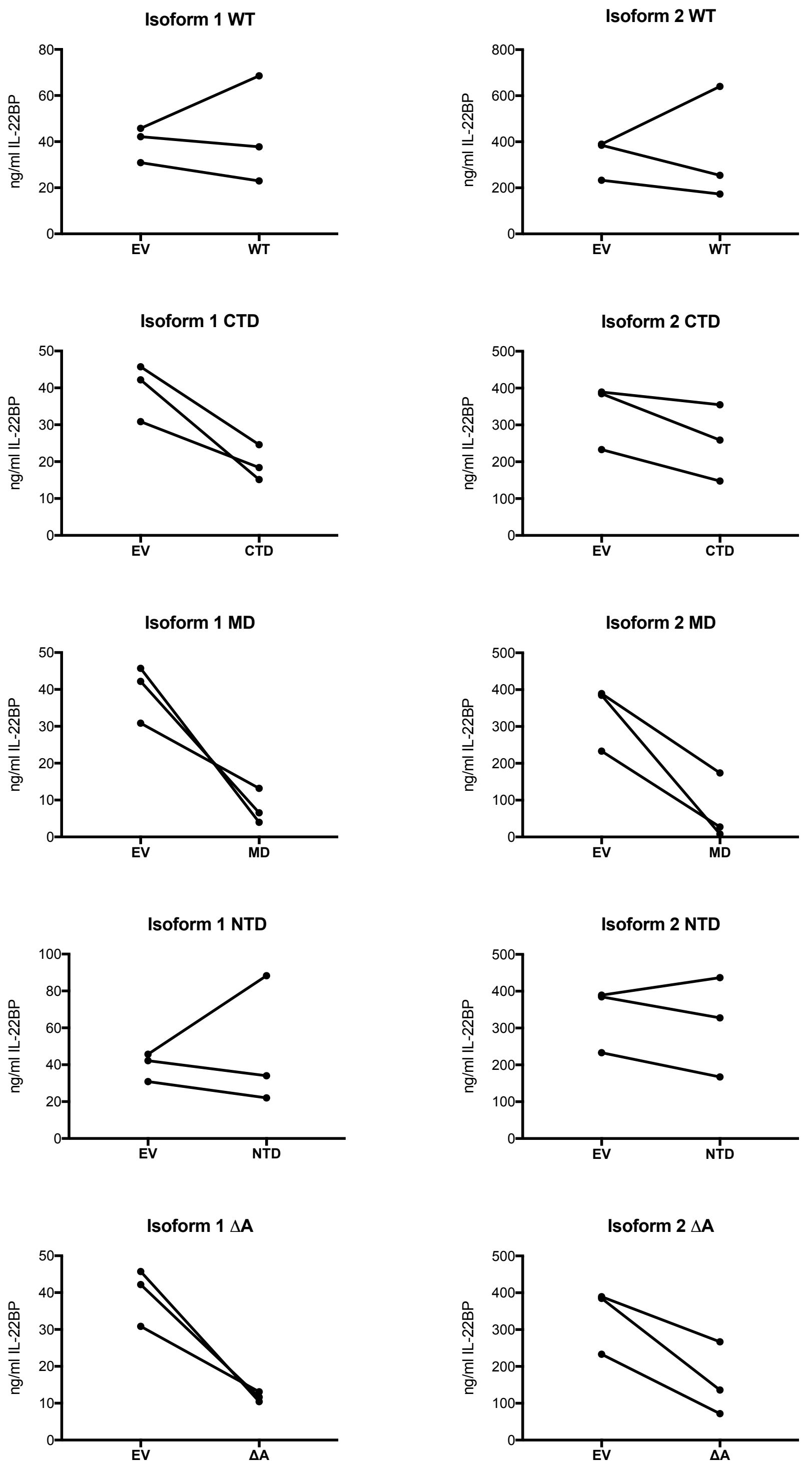

Supplementary Figure 6. Expression vectors for IL-22BP isoform-1 and -2 were individually transfected into HEK293 cells together with either GRP94 wild-type (WT) or GRP94 mutant vectors (CTD, MD, NTD, or deltaA) or empty vector (EV). 24 h after transfection, secreted IL-22BP was analyzed by ELISA.

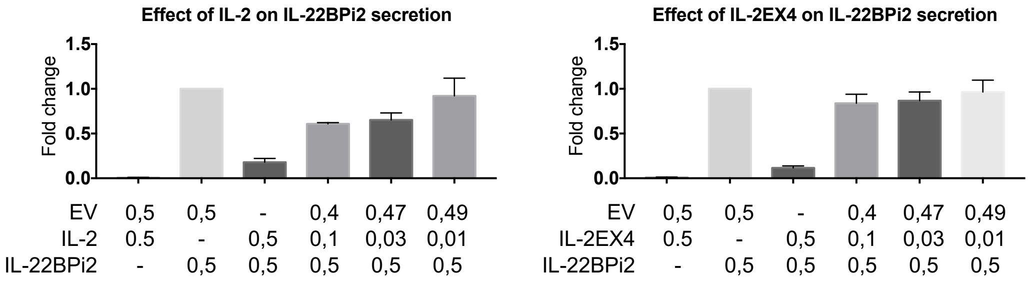

Supplementary Figure 7. IL-22BPi2 secretion is not increased in presence of IL-2 or IL-2EX4. HEK293 cells were co-expressed with different ratios of EV:IL-2:IL-22BPi2 or EV:IL-2EX4:IL-22BPi2 expression plasmids, after 48 hours, IL-22BP secretion was measured by ELISA in conditioned medium. Error bars represent ± SEM of three independent experiments.

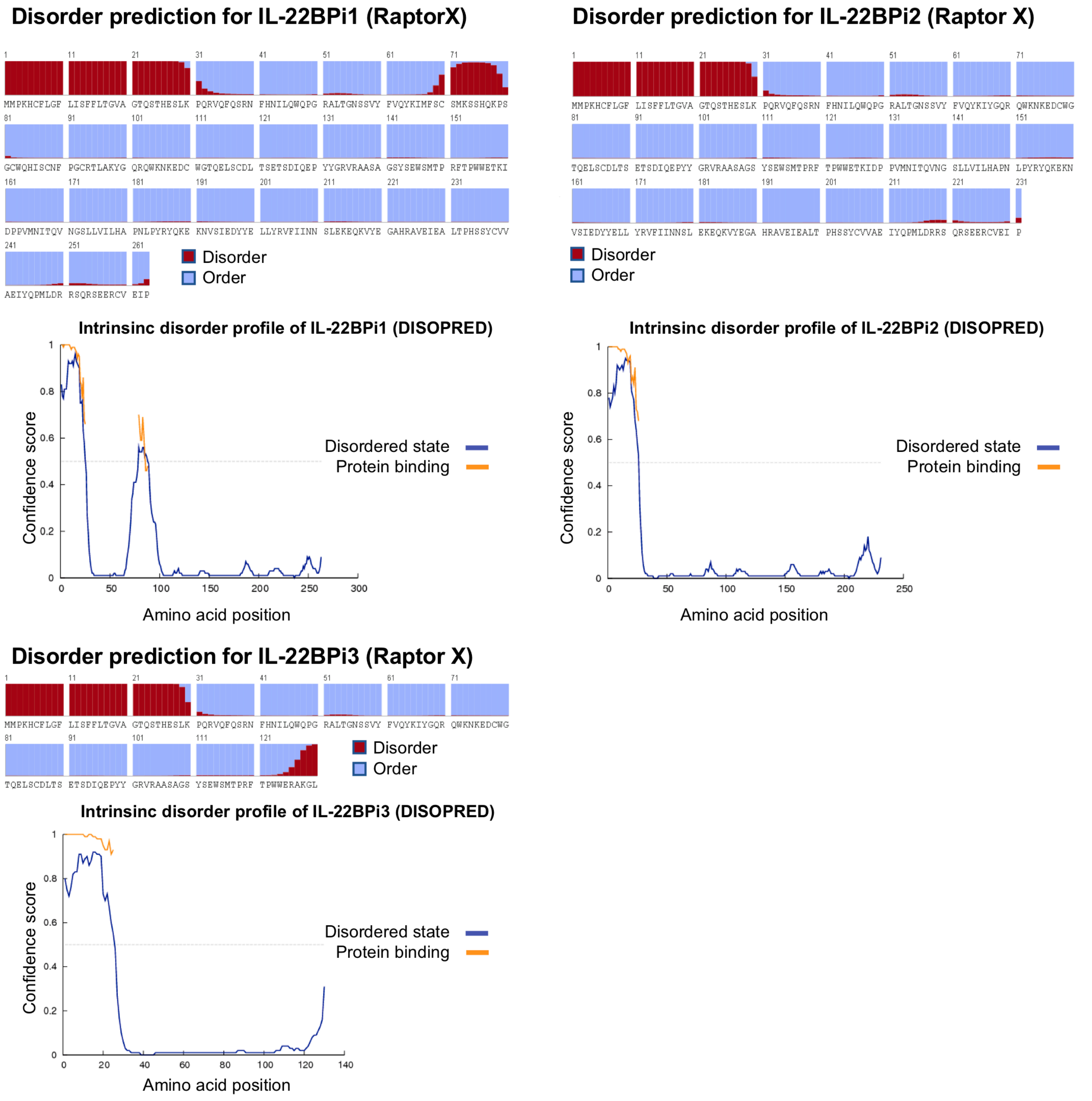

Supplementary Figure 8. IL-22BPi1 is predicted to contain an intrinsic disordered region corresponding to the alternatively spliced exon coding sequence. Two different computational programs for protein disorder prediction were run for the three IL-22BP mature isoforms, i.e. RaptorX (*2*) and DISOPRED3 (*3*).

2.2 Supplementary Tables

Supplementary Table 1. List of IL-22BP antibodies used throughout this study.

IL-22BP antibodies used throughout this study

Nr	Host	Company	Cat Nr
(1)	Mouse	Proteintech	66190
(2)	Rabbit	Abcam	ab133965
(2)	Mouse	Abcam	ab90937
(4)	Goat	R&D	AF1087 and BAF1087

Supplementary Table 2. Primers used for gene expression and cloning.

PRIMERS

- Designed SYBR Green primers (IDT)

Isoform specific	Transcripts	Sequence (5'->3')	Figure
FW IL22RA2	3 *IL22RA2* variants	TGCTTTCTAGGCTTCCTCATC	1A (Gel)
RV IL22RA2		TGAGCCCCTTCATAAACCTT	
FW 1q*	*IL22RA2v1*	ATTTTGCAATGGCAGCCTG	1A (Gel) 1A, 8B, 8C and 8E (qPCR)
RV 1q*		GCCTGGGAAGTTACAAGAAATG	
FW 2&3q*	*IL22RA2v2*	GTGCAGTACAAAATATATGGACAGA	
RV 2q*		GAGGATCTATTTTTGTTTCCCACC	
RV 3q*	*IL22RA2v3*	CTTTTGCTCTTTCCCACCAG	

- Cloning

FW IL22RA2v1 cloning Fragment 1	GCCGCGATCGCCATGATGCCTAAACATTGCTTTCTAG
RV IL22RA2v1 cloning Fragment 1	GCGTCATGCTCCATTCTGAG
FW IL22RA2v1 cloning Fragment 2	CATGTTCTCATGCAGCATGAAAAGCTCTCACCAGA
RV IL22RA2v1 cloning Fragment 2	CGTACGCGTTGGAATTTCCACACATCTCTCTTCACTTC
FW IL2 cloning	CATCGCTCTAGAAATGTTCTCATGCAGCATG
RV IL2 cloning	GACGGCTCTAGACATTTAGCCAATGTTCTGCA

FW GAPDH	CACATCGCTCAGACACCAT	1A, 1D, 2A, 7A, 8B, 8C, 8D, 8E and 8F (qPCR)
RV GAPDH	GCAACAATATCCACTTTACCAGAG	
FW XBP1	TTACGAGAGAAAACTCATGGCC	7C (Gel)
RV XBP1	GGGTCCAAGTTGTCCAGAATGC	

- Pre-designed SYBR Green primers (IDT)

Gene	Oficial name	Reference	
HPRT1	HPRT1	Hs.PT.58v.45621572	8G
IL22RA2 BC (3 *IL22RA2* variants)	IL22RA2	Hs.PT.58.40811	1D, 2A, 8B, 8C, 8D, 8E,8F and 8G (qPCR)
HERP	HERPUD1	Hs.PT.58.21409911	7A and 8G (qPCR)
CHOP	DDIT3	Hs.PT.58.3400360	
GRP78	HSPA5	Hs.PT.58.22715160	
ERP44	ERP44	Hs.PT.58.4529248	
PPIB	PPIB	Hs.PT.58.40291667	

Pre-designed SYBR Green primers (Qiagen)

Gene	Oficial name	Reference	
GRP94	HSP90	QT01848273	7A and 8G (qPCR)
ERDj3	DNAJB11	QT00042560	
GRP170	HYOU1	QT00046214	

TaqMan primers (Thermo)

Gene	Oficial name	Reference	
IL22RA2 (3 *IL22RA2* variants)	IL22RA2	Hs00364814_m1	1B and 1C (qPCR)
IL12B	IL12B	Hs01011519_m1	
IL6	IL6	Hs00174131_m1	
ACTB	ACTB	Hs99999903_m1	

*Taken from ref. #12: Lim C, Hong M, Savan R. Human IL-22 binding protein isoforms act as a rheostat for IL-22 signaling. Sci Signal. 2016 Sep 27;9(447):ra95-ra95. Available from: http://stke.sciencemag.org/cgi/doi/10.1126/scisignal.aad9887

3 References

1. Holden P, Horton WA. Crude subcellular fractionation of cultured mammalian cell lines. BMC Res Notes [Internet]. 2009;2(1):243. Available from: http://bmcresnotes.biomedcentral.com/articles/10.1186/1756-0500-2-243

2. Wang S, Li W, Liu S, Xu J. RaptorX-Property: a web server for protein structure property prediction. Nucleic Acids Res. 2016;44(W1):W430–5.

3. Jones DT, Cozzetto D. DISOPRED3: precise disordered region predictions with annotated protein-binding activity. Bioinformatics [Internet]. 2015 Mar 15;31(6):857–63. Available from: https://academic.oup.com/bioinformatics/article-lookup/doi/10.1093/bioinformatics/btu744
