# Supplementary material for: Long Interleukin-22 Binding Protein Isoform-1 Is an Intracellular Activator of the Unfolded Protein Response
Source: Front Immunol. 2018 Dec 14;9:2934. doi: 10.3389/fimmu.2018.02934 (PMC6302113; doi:10.3389/fimmu.2018.02934)
Supplement: Supplementary Data 1 — Analysis of IL22RA2 and cytokine gene expression by qPCR and surface expression markers by flow cytometry in CD16−/CD14+ or CD16+ monocytes and their corresponding derived immature and mature dendritic cells. [file Data_Sheet_1.PDF]

Supplementary Data 1

Analysis of *IL22RA2* and cytokine gene expression by qPCR and surface expression markers by flow cytometry in $CD16^-/CD14^+$ or $CD16^+$ monocytes and their corresponding derived immature and mature dendritic cells.

We tested whether *IL22RA2* expression patterns were different in distinct monocyte subpopulations stratified according to $CD14^+$ and $CD16^+$ expression. The approach was based on an initial granulocyte and NK cell depletion, followed by $CD16^+$ selection (Miltenyi, 130-091-765) of both non-classical and intermediate monocytes, while the CD16-negative fraction was subjected to a further CD14-positive selection (Miltenyi, 130-050-201) consisting of classical monocytes. Flow cytometry graphs and scheme of this strategy are presented below (**Figure 1**).

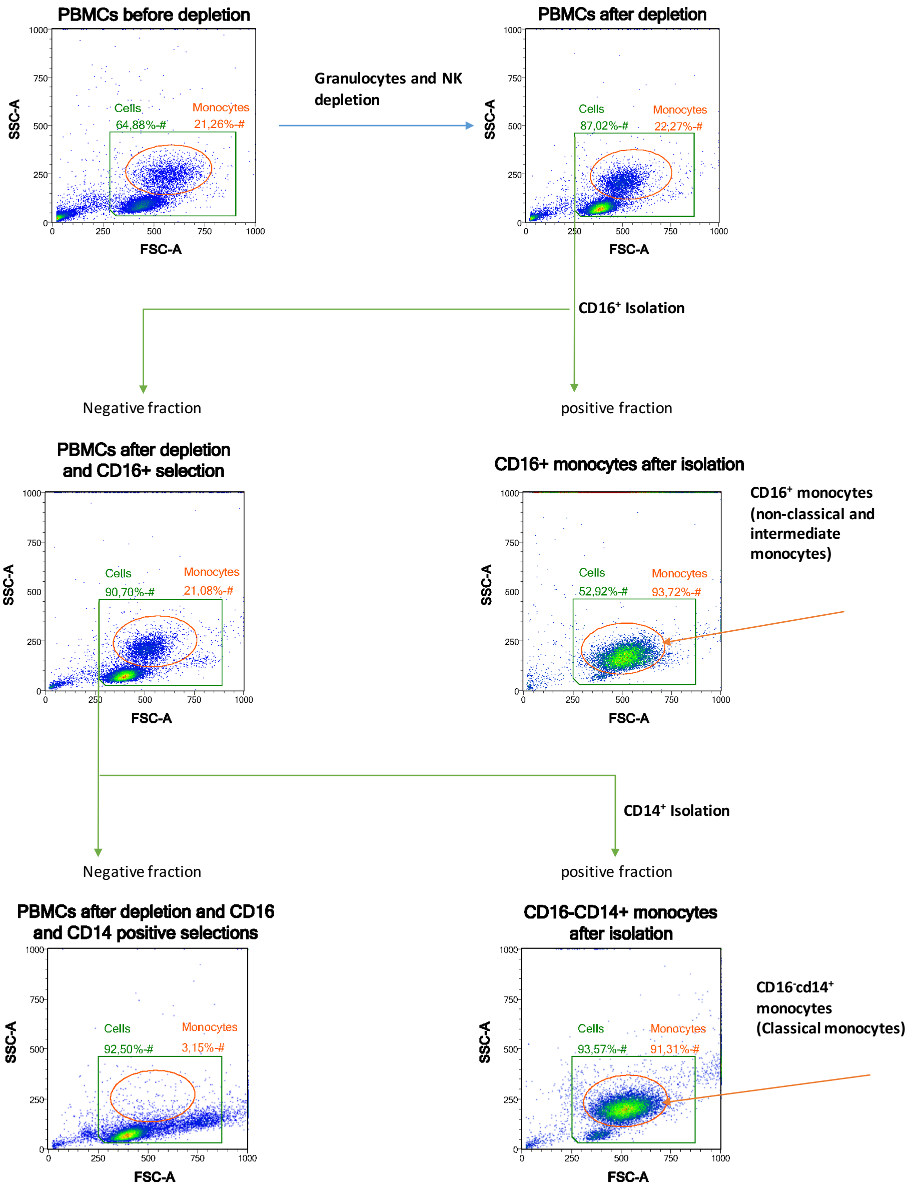

Figure 1: $CD16^-/CD14^+$ and $CD16^+$ monocyte purification analysed by flow cytometry.

In order to generate immature and mature moDCs, we cultured the purified monocytes in Mo-DC medium and matured them by treatment with IFN-γ and then with LPS for 24h or left untreated (immature) as described in **Materials and Methods** section. Assessment of expression of surface maturation markers before and after maturation was done by flow cytometry and is summarized in the following **Figure 2**.

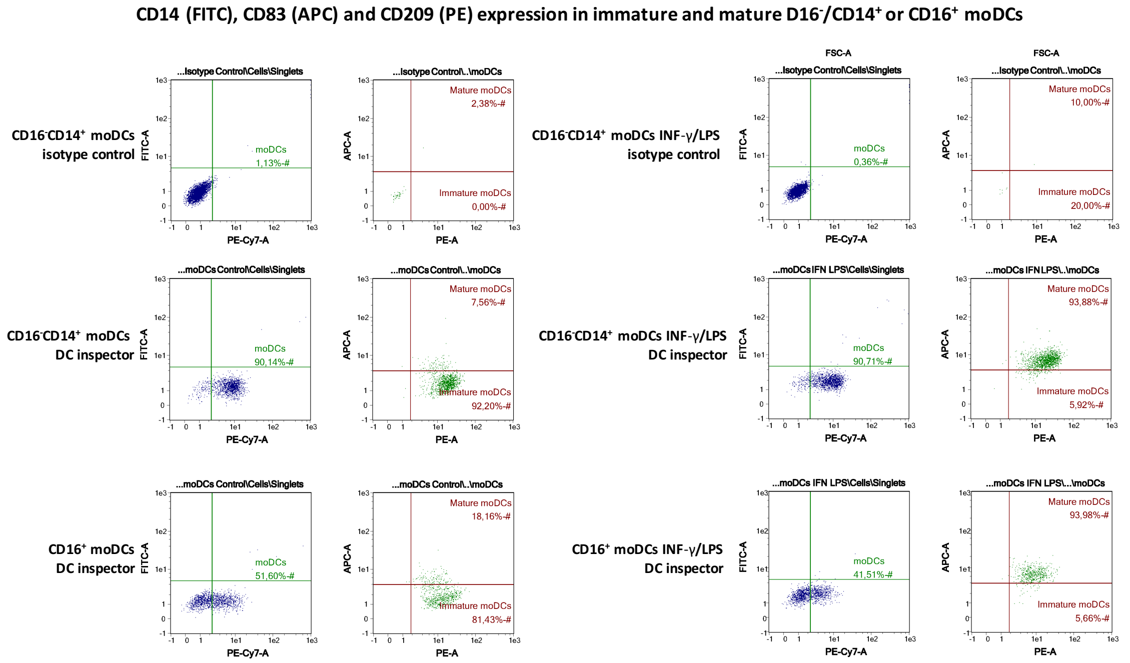

Figure 2: Flow cytometric analysis of in vitro generated monocyte-derived dendritic cells (moDCs) using the Mo-DC Differentiation Inspector. Surface markers (CD14, CD83 and CD209) expression of CD16⁻/CD14⁺ or CD16+ moDCs were analysed. CD14=FITC; CD83=APC and CD209=PE.

We analysed expression of cytokine genes at day 0 (freshly isolated monocytes) and at day 7 (immature and matured moDCs) (**Figure 3**). Primers and probes are indicated at the end of this supplement.

IL22RA2 levels were higher in CD16⁺ compared to CD14⁺ monocytes, while *IL10* and *IL6* followed opposite trends (**Figure 3**, left). However, expression of *IL22RA2*

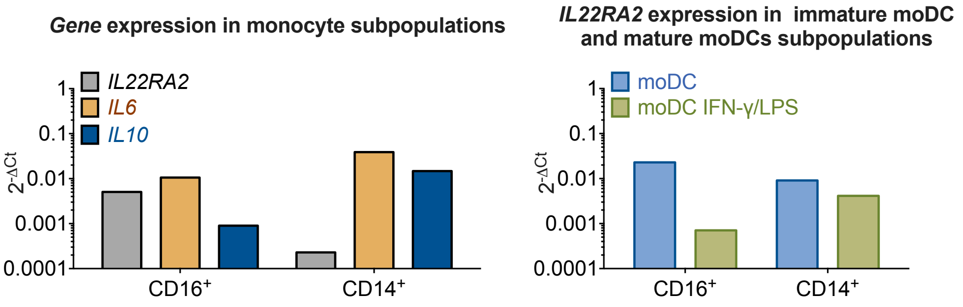

Figure 3: IL22RA2 and cytokine gene expression in CD16⁻/CD14⁺ or CD16⁺ monocytes and in their corresponding immature and mature monocyte derived dendritic cells (moDCs) subpopulations.

increased to similar levels upon cultivation of CD16⁺ or CD14⁺ monocytes in Mo-DC medium (**Figure 3**, right). Maturation decreased *IL22RA2* expression in both subpopulations; nevertheless, the decrease was marked more strongly in CD16⁺-monocyte derived, compared to CD14⁺-monocyte derived DCs (**Figure 3**, right).

We further analyzed more extensive cytokine expression signatures in addition to *IL22RA2* in the corresponding moDCs. In both CD16$^+$ monocyte-derived and CD14$^+$ monocyte-derived immature DCs, we observed similar expression levels under non-stimulated conditions for *IL23A*, *IL18*, *IFNB*, *IL12B*, *IL10* and *IL20*. However, *IL22RA2* levels were modestly higher (2,5-fold increase) in CD16$^+$ moDCs, while *IL19* levels were much higher (17-fold increase) in CD14$^+$ moDCs.

When comparing IFN-γ/LPS-stimulated mature moDCs with immature moDCs (**Table 1**), a strong induction of *IL12B* (about 120 fold increase) as well as smaller ones of *IL18* (about 8-fold increase) and *IL20* (about 2-fold increase) were observed in both CD14$^+$ and CD16$^+$ derived populations. *TGFβ* was down-regulated (about 3-fold decrease) to similar extent in both populations. However, *IL22RA2* showed a 3-fold decrease in CD16$^-$CD14$^+$ moDCs and a 32-fold decrease in CD16$^+$ moDCs. Various other cytokines were differentially upregulated during culture with the maturation stimuli IFN-γ/LPS. In CD16$^-$CD14$^+$ moDCs compared to CD16$^+$ moDCs, *IFNB* (28-fold increase vs. no difference) and *IL23A* (17-fold increase vs. 5) showed higher levels of upregulation, while *IL19* (2-fold change vs. 91) and *IL10* (7-fold change vs. 41) were less strongly upregulated.

Table 1: Cytokine gene expression fold-change of mature moDCs relative to immature moDCs.

	**CD14$^+$-monocyte derived DCs treated on D7 with IFN-γ / LPS**	**CD16$^+$-monocyte derived DCs treated on D7 with IFN-γ / LPS**
IL12B	124	114
IL19	2	91
IL10	7	41
IFNB	28	1,24
IL23A	17	5
IL18	7	9
IL20	2	1,5
IL22RA2	2	32
TGFB	3	4

(dark green)	Highly increased (>10)
(light green)	Moderately increased (<10)
(dark red)	Moderately decreased (<10)
(red)	Highly decreased (>10)

Based on these findings, our research continued in identifying the CD16$^+$ monocyte subpopulation (intermediate, non-classical or both) that expressed higher levels of *IL22RA2*, described in the main article text, but we did not further focus on the effects of maturation nor on the other cytokine genes in these 3 populations.

As shown in **Figure 8** in the main article text, we noted that although *IL22RA2* levels are monocyte-subpopulation specific, following *in vitro* culture in Mo-DC differentiation medium, these differences disappear, and therefore, CD14$^+$ moDCs are a suitable model for the study of *IL22RA2*.

Primers and probes used in Supplementary Data 1.

Gene	Assay ID	Type	Dye	Catalog #	Company
IFNB	Hs01077958_s	Probe	FAM	4331182	Thermo Fisher
IL12B	Hs01011519_m1	Probe	FAM	4331182	Thermo Fisher
IL23A	Hs00900828_g1	Probe	FAM	4331182	Thermo Fisher
IL6	Hs00174131_m1	Probe	FAM	4331182	Thermo Fisher
IL19	Hs00604657_m1	Probe	FAM	4331182	Thermo Fisher
IL10	Hs00961622_m1	Probe	FAM	4331182	Thermo Fisher
IL20	Hs00218888_m1	Probe	FAM	4331182	Thermo Fisher
IL22RA2	Hs00364814_m1	Probe	FAM	4331182	Thermo Fisher
ACTB	Hs99999903_m1	Probe	VIC	4448489	Thermo Fisher
IL18	Hs.PT.58.25675872	Primers	-	-	IDT
TGFB	Hs. PT.58.22563137	Primers	-	-	IDT
IL22RA2	Hs.PT.58.40811	Primers			IDT
ACTB	Hs. PT.39a.22214847	Primers			IDT
